# A Viral Satellite RNA Induces Yellow Symptoms on Tobacco by Targeting a Gene Involved in Chlorophyll Biosynthesis using the RNA Silencing Machinery

**DOI:** 10.1371/journal.ppat.1002021

**Published:** 2011-05-05

**Authors:** Hanako Shimura, Vitantonio Pantaleo, Takeaki Ishihara, Nobutoshi Myojo, Jun-ichi Inaba, Kae Sueda, József Burgyán, Chikara Masuta

**Affiliations:** 1 Research Faculty of Agriculture, Hokkaido University, Kita-ku, Sapporo, Japan; 2 Istituto di Virologia Vegetale, CNR, Torino, Italy; University of California, Riverside, United States of America

## Abstract

Symptoms on virus-infected plants are often very specific to the given virus. The molecular mechanisms involved in viral symptom induction have been extensively studied, but are still poorly understood. *Cucumber mosaic virus* (CMV) Y satellite RNA (Y-sat) is a non-coding subviral RNA and modifies the typical symptom induced by CMV in specific hosts; Y-sat causes a bright yellow mosaic on its natural host *Nicotiana tabacum*. The Y-sat-induced yellow mosaic failed to develop in the infected *Arabidopsis* and tomato plants suggesting a very specific interaction between Y-sat and its host. In this study, we revealed that Y-sat produces specific short interfering RNAs (siRNAs), which interfere with a host gene, thus inducing the specific symptom. We found that the mRNA of tobacco magnesium protoporphyrin chelatase subunit I (*ChlI*, the key gene involved in chlorophyll synthesis) had a 22-nt sequence that was complementary to the Y-sat sequence, including four G-U pairs, and that the Y-sat-derived siRNAs in the virus-infected plant downregulate the mRNA of *ChlI* by targeting the complementary sequence. *ChlI* mRNA was also downregulated in the transgenic lines that express Y-sat inverted repeats. Strikingly, modifying the Y-sat sequence in order to restore the 22-nt complementarity to *Arabidopsis* and tomato *ChlI* mRNA resulted in yellowing symptoms in Y-sat-infected *Arabidopsis* and tomato, respectively. In 5′-RACE experiments, the *ChlI* transcript was cleaved at the expected middle position of the 22-nt complementary sequence. In GFP sensor experiments using agroinfiltration, we further demonstrated that Y-sat specifically targeted the sensor mRNA containing the 22-nt complementary sequence of *ChlI*. Our findings provide direct evidence that the identified siRNAs derived from viral satellite RNA directly modulate the viral disease symptom by RNA silencing-based regulation of a host gene.

## Author Summary

*Cucumber mosaic virus* (CMV) Y satellite RNA (Y-sat) is an interesting subviral RNA because it changes the green mosaic induced by CMV into a bright yellow mosaic in *Nicotiana tabacum.* The molecular basis underlying the induction of symptoms by viruses is not well understood, and this Y-sat-mediated modification of symptoms has been a long-standing mystery. In this study, we discovered the molecular mechanism involved in the Y-sat-induced yellowing. First, we showed that transgenic *N*. *benthamiana* plants that expressed the inverted-repeat sequence of Y-sat also developed a yellow phenotype, similar to the Y-sat-infected plants. Then, we found that tobacco magnesium protoporphyrin chelatase subunit I gene (*ChlI*, the key gene involved in chlorophyll synthesis) was downregulated in the transgenic plants and in the Y-sat-infected plants. We then identified a 22-nt long sequence that is complementary to the Y-sat including four G-U pairs in the *ChlI* mRNA. Finally, we demonstrated that a short interfering RNA (siRNA) derived from Y-sat specifically targeted and downregulated the *ChlI* mRNA, thus impairing the chlorophyll biosynthesis pathway. This discovery of the molecular basis of the symptom modification induced by Y-sat is the first demonstration that a subviral RNA can induce disease symptoms by regulating host gene expression through the RNA silencing machinery.

## Introduction

Plants infected with viruses often display various symptoms, which can be very specific to given viruses. Despite past efforts, the molecular bases underlying virus-induced diseases symptoms are still poorly understood. Subviral non-coding RNA molecules such as satellite RNAs (satRNAs) or defective interfering (DI) RNAs are often associated with plant viruses and can modify the symptoms induced by helper viruses [1], [2], [3]. Because such subviral RNAs dramatically modify the symptoms induced by helper viruses, they are potential tools for gaining insights into the molecular mechanisms of symptom development.

SatRNAs of *Cucumber mosaic virus* (CMV) are dependent on helper viruses for their replication and encapsidation and often attenuate the disease symptoms induced by CMV. Specifically, Y-satellite RNA (Y-sat) modifies the symptoms and exacerbates the pathogenicity of CMV in specific hosts; Y-sat induces a bright yellowing of leaves of *Nicotiana tabacum* (the natural host) and other related species (i.e., *N*. *benthamiana*), which is yellower than a typical chlorosis, whereas it induces systemic necrosis on tomato [4], [5], [6], [7]. The sequence domains on Y-sat, which are responsible for the symptom induction, have been identified in our previous and several other reports [6], [7], [8], [9], [10]. We also suggested that a single, nuclear-encoded, incompletely dominant gene in tobacco controls the Y-sat-mediated yellowing in tobacco plants [11], but no such host genes have ever been shown to be involved in the symptom modification nor has the molecular mechanism been reported. An attractive model based on RNA silencing has been suggested [2], [12], but the solid experimental data are still needed.

RNA silencing is a conserved, sequence-specific gene regulation system, which has an essential role in development and maintenance of genome integrity. RNA silencing relies on short RNA (sRNA) molecules (21–24 nt), which are the key mediators of RNA silencing-related pathways in almost all eukaryotic organisms [13], [14], [15]. In plants, similar to other eukaryotic organisms, there are two main classes of sRNAs: microRNAs (miRNAs) and short interfering RNAs (siRNAs), but the latter class contains several different types [16], [17]. These sRNAs are produced from double-stranded RNA (dsRNA) or from folded structures by Dicer-like (DCL) proteins and guide Argonaute (AGO) proteins to target cognate RNA or DNA sequences [13], [18]. In higher plants, RNA silencing also operates as an adaptive inducible antiviral defense mechanism. As a counter-defense strategy, plant viruses have evolved viral suppressors of RNA silencing (VSRs) [19] that interfere with the RNA silencing pathway at different steps by binding to viral siRNA and/or dsRNAs or directly interacting with AGO1 [20], [21].

Subviral RNAs such as satRNA and DI RNA of tombusvirus have been also used to understand the roles of RNA silencing in viral replication and in symptom development. The DI RNA-induced RNA silencing response is known to control the level of helper virus, facilitating the long-term co-existence of the host and the viral pathogen [20], [22], [23], [24]. In addition, progress in understanding plant antiviral RNA silencing has revealed cross relationships between RNA silencing and viral pathogenicity. Recent studies suggest the possibility that virus-derived siRNA (vsiRNA) could mediate virus–host interactions through a shared sequence identity with the host mRNA, resulting in silencing of the host genes and subsequent viral symptom development. A few interactions between host mRNAs and vsiRNAs that resulted in the vsiRNA-guided cleavages of host mRNAs have been experimentally shown [25], [26], although their roles in the virus–host interaction have not been determined to date.

Magnesium (Mg)-chelatase is the key enzyme in chlorophyll biosynthesis, and three subunits (ChlI, ChlH and ChlD) of the tobacco magnesium protoporphyrin chelatase are required for the proper function of the enzyme [27]. Indeed, tobacco plants defective for *ChlI* have the yellow phenotype [28], suggesting that chlorophyll biosynthesis is impaired. The same yellow phenotype was observed when the *ChlI* gene of tobacco or cotton was targeted by virus-induced gene silencing (VIGS) [29], [30], [31]. Furthermore, an *Arabidopsis* mutant defective for *ChlI* also had pale-green to yellow leaves [32]. Importantly, the plants defective in the function of the Mg-chelatase enzyme had a very similar yellow phenotype to plants infected with CMV and Y-sat. Thus, these results raised the possibility that the *ChlI* is downregulated by Y-sat in the virus-infected plants.

In this study, we show that transgenic *N*. *benthamiana* plants develop a yellow phenotype when expressing the inverted-repeat sequence of Y-sat, similar to the symptoms of the Y-sat-infected plants. Moreover, we provided evidence that Y-sat targets the *ChlI* gene using the host RNA silencing machinery in such a way that Y-sat-derived siRNAs efficiently downregulate *ChlI* mRNA through RNA silencing-mediated cleavage. Our findings strongly suggest that this yellow phenotype is the result of a disorder in chlorophyll synthesis caused by the downregulation of the *ChlI* gene.

## Results

### Biosynthesis of chloroplast pigments is impaired in *Nicotiana benthamiana* plants that express the dsRNA of Y-sat

**Figure 1:**
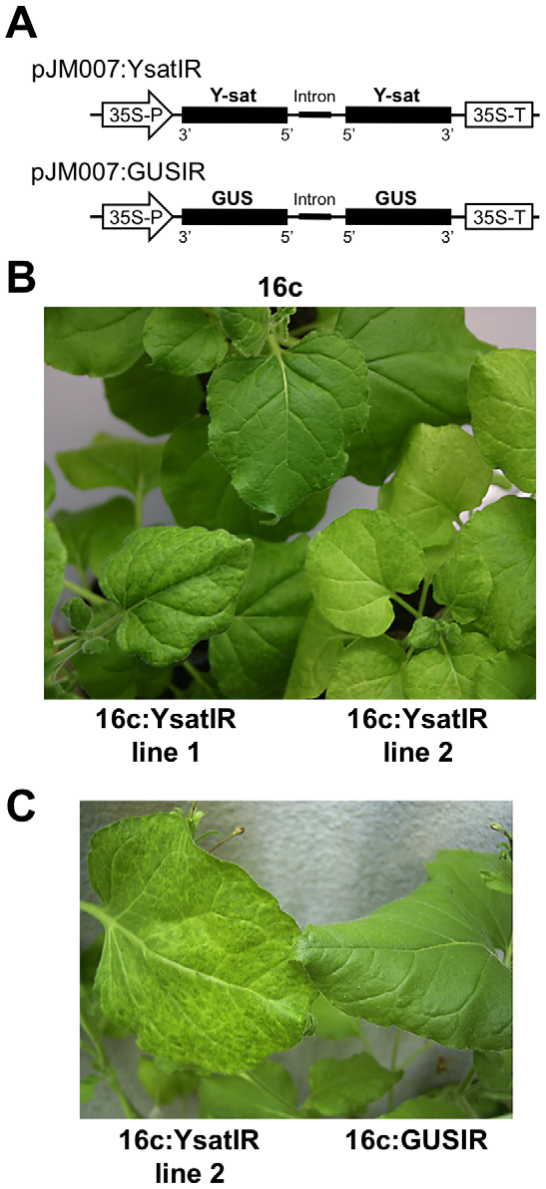
*Nicotiana benthamiana* plants expressing the dsRNA of Y-sat have a yellow phenotype without viral infection. A. Schematic representation of the vector construct for expressing the dsRNA sequence of Y-sat or GUS. The 317-bp (53 to 369) Y-sat sequence was inserted in pJM007 vector [46] in a head-to-head manner to create Y-sat dsRNA, then the inverted repeat (IR)-expressing cassette was transferred to Ti-plasmid vector pIG121-Hm. The 1004-nt dsRNA of the GUS sequence (GUS) was used as a control. 35S-P, *Cauliflower mosaic virus* (CaMV) 35S promoter; 35S-T, terminator of CaMV 35S promoter. The intron is derived from IV2 of the *ST-LS1* gene from tobacco [46]. B. Transgenic *N*. *benthamiana* plants that express the inverted repeat of Y-sat had either the vein yellowing (16c:YsatIR line 1) or yellow mosaic phenotype (16c:YsatIR line 2); the control *N*. *benthamiana* had the typical green phenotype (16c). These plants were the same age and had been grown together in the same conditions. C. Transgenic *N*. *benthamiana* 16c plant that expresses the inverted repeat of GUS. The yellow phenotype was not observed on *N*. *benthamiana* expressing dsRNA of GUS (16c:GUSIR), while 16c:YsatIR line 2 had vein yellowing. These plants were the same age and had been grown together in the same conditions.

To identify host genes involved in the Y-sat-induced symptom modification, we created transgenic *N. benthamiana* plants that express the Y-sat sequence, expecting the yellow phenotype to be induced without CMV as a helper virus. We have used this strategy to avoid any effect of virus replication on host gene expression, because virus infection itself has been shown to regulate the expression of numerous genes [33]. We first created transgenic plants that expressed the Y-sat sequence either in the sense or antisense orientation, but these transgenic plants failed to have any phenotypic changes (data not shown). However, when the Y-sat inverted-repeat (IR) sequence-expressing cassette (Figure 1A) was introduced into *N*. *benthamiana* plants, we observed that the transgenic *N*. *benthamiana* lines (16c:YsatIR) had a yellow phenotype (Figure 1B), although the yellow phenotype was less pronounced in the 16c:YsatIR lines than in the Y-sat-replicating system. Of four transgenic lines that we obtained, two had phenotypes with distinct yellowing; line 1 had vein yellowing, and line 2 had a yellow mosaic. No yellow phenotype was observed on the *N*. *benthamiana* that expressed dsRNA of GUS (16c:GUSIR), demonstrating that the expression of dsRNA of an unrelated sequence in the same Y-sat IR transformation cassette does not cause a yellow symptom (Figure 1B and 1C). We also confirmed the lack of viral contamination in the 16c:YsatIR lines by RT-PCR using primers that are specific to CMV genomes (data not shown).

To identify putative plant genes responsible for the yellow phenotype, we carried out microarray analyses of RNA extracted from the 16c:YsatIR plants (Text S1). In 16c:YsatIR plants, 134 genes were significantly downregulated to levels that are at least 40% lower than in their wild-type counterparts (*N*. *benthamiana* 16c) (Table S1). Among them, 31 genes were actually involved in chlorophyll biosynthesis and chloroplast biogenesis (Table S1), further supporting the hypothesis that the yellow phenotype could be the result of downregulation of the host gene(s) involved in the biosynthesis pathway of chloroplast pigments. Indeed, proteome analyses showed that several chloroplast-related proteins, such as RuBisCo small subunit, RuBisCo activase and glyceraldehyde-3-phosphate dehydrogenase were significantly affected in 16c:YsatIR plants (Text S1, Figure S1). More interestingly, the mobility of the RuBisCo small subunits was shifted in a two-dimensional gel (Figure S1), indicating that the proteins had been modified. All together, these results suggest that the expression of chloroplast-related genes and subsequent synthesis of proteins were altered in the 16c:YsatIR plants.

### *ChlI* mRNA is downregulated in 16c:YsatIR plants, in *N. benthamiana* plants infected with Y-sat, and in Y-sat dsRNA-transfected protoplasts

**Figure 2:**
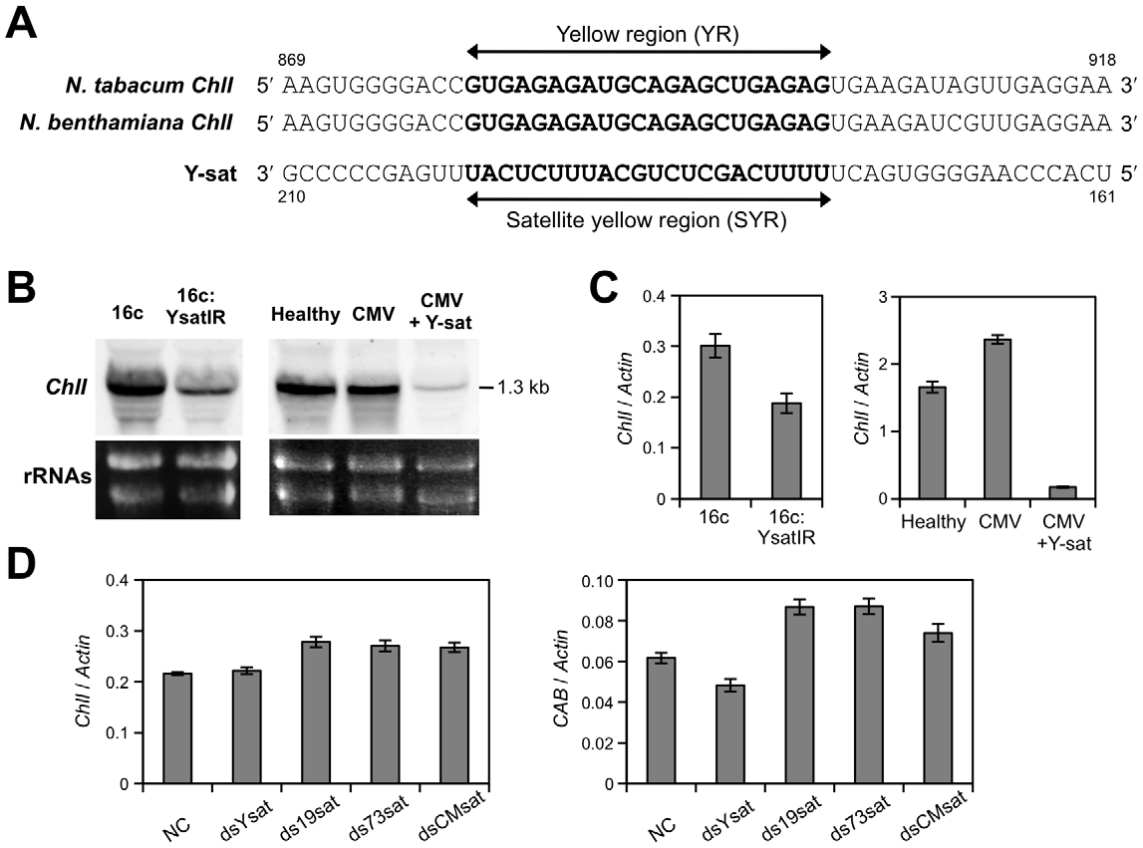
The tobacco magnesium protoporphyrin chelatase subunit (*ChlI*) gene, which has the 22-nt complementary sequence to the Y-sat sequence, was downregulated in the presence of Y-sat. A. The 22-nt complementary sequences of the *ChlI* gene of *N*. *tabacum*, *N*. *benthamiana* and Y-sat. The 22-nt complementary sequences in *ChlI* (yellow region; YR) and in the Y-sat (satellite yellow region; SYR) are in bold face. The 22-nt complementary sequences include four G-U base pairs. B. Northern hybridization of *ChlI* mRNA of the 16c:YsatIR and Y-sat-infected plants. Total RNAs were prepared from 16c:YsatIR, and CMV-infected *N*. *benthamiana* with or without Y-sat. As a helper virus, CMV strain Y was used. The levels of *ChlI* mRNA in healthy *N*. *benthamiana* (wild type and 16c) were also examined. The 371-bp DIG-labeled cDNA probe was synthesized from the 3′ region of the *ChlI* gene of *N*. *benthamiana*. Ribosomal RNAs were used as a loading control (lower panel). C. The mRNA levels of *ChlI* determined by quantitative real-time RT-PCR. Total RNAs were prepared from 16c:YsatIR and CMV-infected *N*. *benthamiana* with or without Y-sat. As a helper virus, CMV strain Y was used. The *ChlI* mRNA levels in healthy *N*. *benthamiana* (wild type and 16c) were also examined. The *ChlI* mRNA levels relative to the *actin* mRNA level are shown (mean ± SE; *n* = 3). In panels B and C, samples were taken from equivalent leaves of plants grown in the same conditions. D. The mRNA levels of the *ChlI* gene and *CAB* gene in protoplasts after the introduction of dsRNA of Y-sat. Protoplasts prepared from *N*. *benthamiana* (wild type) leaves were transfected with each dsRNA (2 µg) of Y-sat, S19-sat, T73-sat and CM-sat (dsYsat, dsS19sat, dsT73sat, dsCMsat, respectively). Protoplasts were harvested at 20 h after transfection, and the mRNA levels of the *ChlI* and *CAB* gene were measured by quantitative real-time RT-PCR (mean ± SE; *n* = 3). The *actin* mRNA levels were used for data normalization. NC; Protoplasts transfected with 2 µL sterile deionized water instead of satRNA.

When we aligned the sequences of the 31 genes involved in chlorophyll biosynthesis and chloroplast biogenesis identified by microarray analysis with the Y-sat sequence, we found a high degree of sequence complementarity (22 nt in a row including four G-U pairs) between the yellow-inducing domain of Y-sat [7], [34] and the tobacco magnesium (Mg) protoporphyrin chelatase subunit I (*ChlI*) gene (accession AF014053). Because ChlI is a component of the primary enzyme that catalyzes the first step in chlorophyll synthesis via the tetrapyrrole biosynthesis pathway [32], this evidence encouraged us to clone and sequence the *ChlI* gene of *N*. *benthamiana*. We then found that both the *ChlI* genes from *N*. *tabacum* and *N*. *benthamiana* had the 22-nt sequence complementary to the Y-sat sequence (Figure 2A). Hereafter, we called the 22-nt complementary sequence for the *ChlI* gene and the Y-sat sequence as the yellow region (YR) and satellite yellow region (SYR), respectively (Figure 2A). We then examined the mRNA levels of the *ChlI* gene by Northern blot analysis and quantitative real-time RT-PCRs in 16c:YsatIR and Y-sat-infected *N*. *benthamiana* plants. The outputs of these analyses showed that the *ChlI* mRNA was markedly downregulated in both plants (Figure 2B and C) and confirmed the results of the microarray analysis. To confirm that the downregulation of the *ChlI* mRNA was due to the satRNA itself, we further conducted a quantitative real-time RT-PCR using RNAs from *N*. *benthamiana* protoplasts transfected with the dsRNA of Y-sat. As controls, we transfected protoplasts with dsRNA of three other CMV satRNAs; S19-sat, T73-sat [35] and CM-sat [36]. These satRNAs are different from Y-sat in the corresponding SYR sequences and do not induce any yellow phenotypes in tobacco plants [35]. As shown in Figure 2D, the *ChlI* mRNA level was lower in protoplasts treated with dsRNA of Y-sat than in those treated with dsRNA of the other satRNAs. In addition, the mRNA level of another chloroplast-related gene, *CAB3*, decreased in the Y-sat dsRNA-treated protoplasts (Figure 2D), confirming our findings from the microarray analysis. In the proteome analysis, many chloroplast-related proteins were affected in the transgenic 16c plants expressing Y-sat dsRNA; thus, it is conceivable that the down-regulation of the *ChlI* gene caused a decrease in other chloroplast-related genes expression in the Y-sat dsRNA-treated protoplasts.

### CMV vector-based gene silencing of the *ChlI* induces downregulation of the *ChlI* mRNA and the yellow symptom

**Figure 3:**
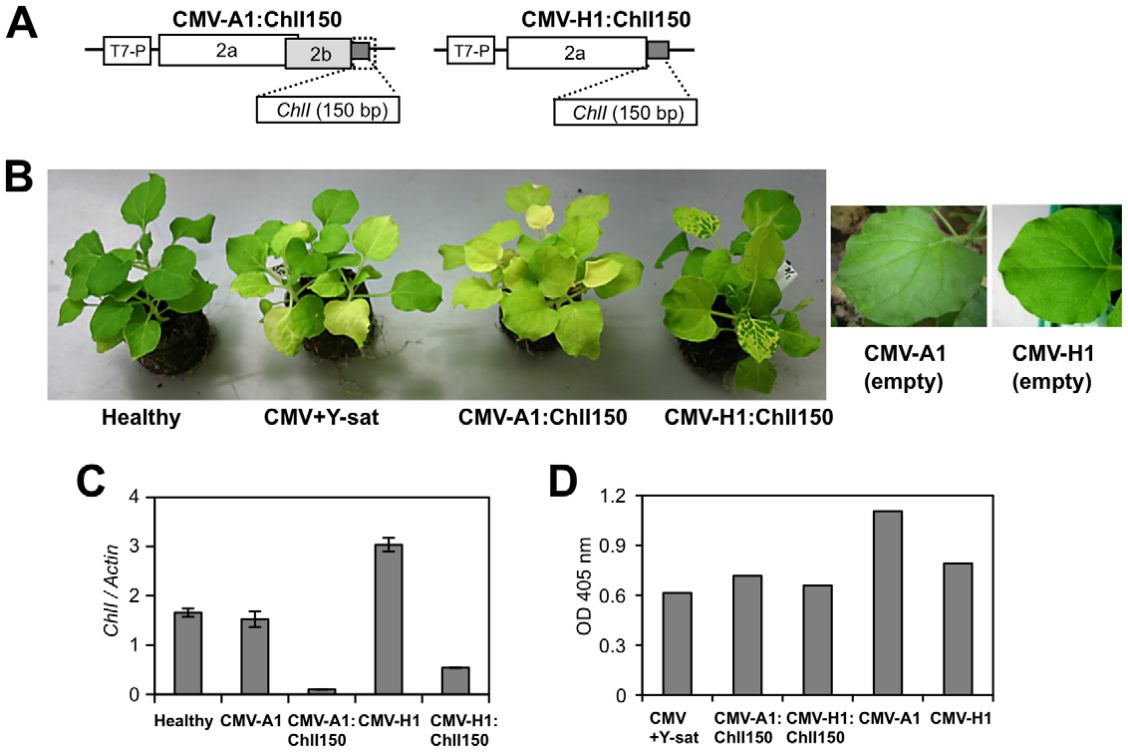
Virus-induced gene silencing of the *ChlI* gene by the CMV vector resulted in yellow symptoms similar to the Y-sat-mediated yellow phenotype. A. Schematic representation of the CMV vector constructs. Both CMV-A1 and CMV-H1 are derived from RNA2 of CMV-Y. CMV-A1 lacks the C-terminal one-third of the intact 2b protein, while CMV-H1 vector lacks the entire 2b protein. The 150-bp of the *ChlI* gene (817 to 966) was inserted into the two CMV vectors. T7-P, T7 promoter. B. Yellow phenotypes of the *N*. *benthamiana* plants infected with CMV vector carrying the sequence of the *ChlI* gene. Empty vectors did not induce any yellow symptoms in the systemically infected leaves, but CMV-A1:ChlI150 and CMV-H1:ChlI150 induced systemic yellow symptoms (yellow mosaic and vein yellowing) similar to those induced by Y-sat. To avoid severe symptoms, we used a pseudorecombinant virus that contains RNA components derived from RNA1 and RNA3 of CMV strain L. C. The mRNA levels of the *ChlI* gene in the CMV-infected tissues determined by quantitative real-time RT-PCR. *ChlI* mRNA levels relative to the *actin* mRNA level are shown (mean ± SE; *n* = 3). RNAs were extracted from systemic leaves infected with the virus shown in panel B at 9 dpi. D. Virus levels in the systemic leaves of *N*. *benthamiana* inoculated with the CMV vector at 14 dpi, which were determined by conventional ELISA using antibodies raised against CMV CP. Samples are those in panel B.

We next examined whether silencing of the *ChlI* gene using VIGS can induce similar yellow symptoms in the absence of Y-sat. The 150-bp of *ChlI* (817 to 966) was inserted into the two CMV vectors, CMV-A1 and CMV-H1; CMV-A1 lacks the C-terminal one-third of the intact 2b protein [37], while CMV-H1 vector lacks the entire 2b protein [38] (Figure 3A). In the VIGS experiments, we used a pseudorecombinant virus that contains RNA components derived from RNA1 and RNA3 of CMV strain L to avoid the severe mosaic symptoms induced by CMV-Y. *N. benthamiana* plants infected with either of the viral vectors had systemic yellow symptoms similar to those induced by the replicating Y-sat in the presence of the helper virus (Figure 3B). Although CMV-H1:ChlI150 induced the yellowing more slowly than CMV-A1:ChlI150 in the early stage of infection, the results of quantitative real-time RT-PCR confirmed that the *ChlI* mRNA was downregulated in both CMV-A1:ChlI150- and CMV-H1:ChlI150-infected *N. benthamiana* plants compared to control plants infected with one of the empty vectors (Figure 3C). Using enzyme-linked immunosorbent assay (ELISA), we confirmed that both pseudorecombinant viruses carrying the inserted *ChlI* sequence replicated and accumulated to a similar level in the systemic leaves at 14 days post-inoculation (dpi) (Figure 3D).

### Sequence complementarity between Y-sat and the ChlI gene is essential for the induction of the yellow phenotype

**Figure 4:**
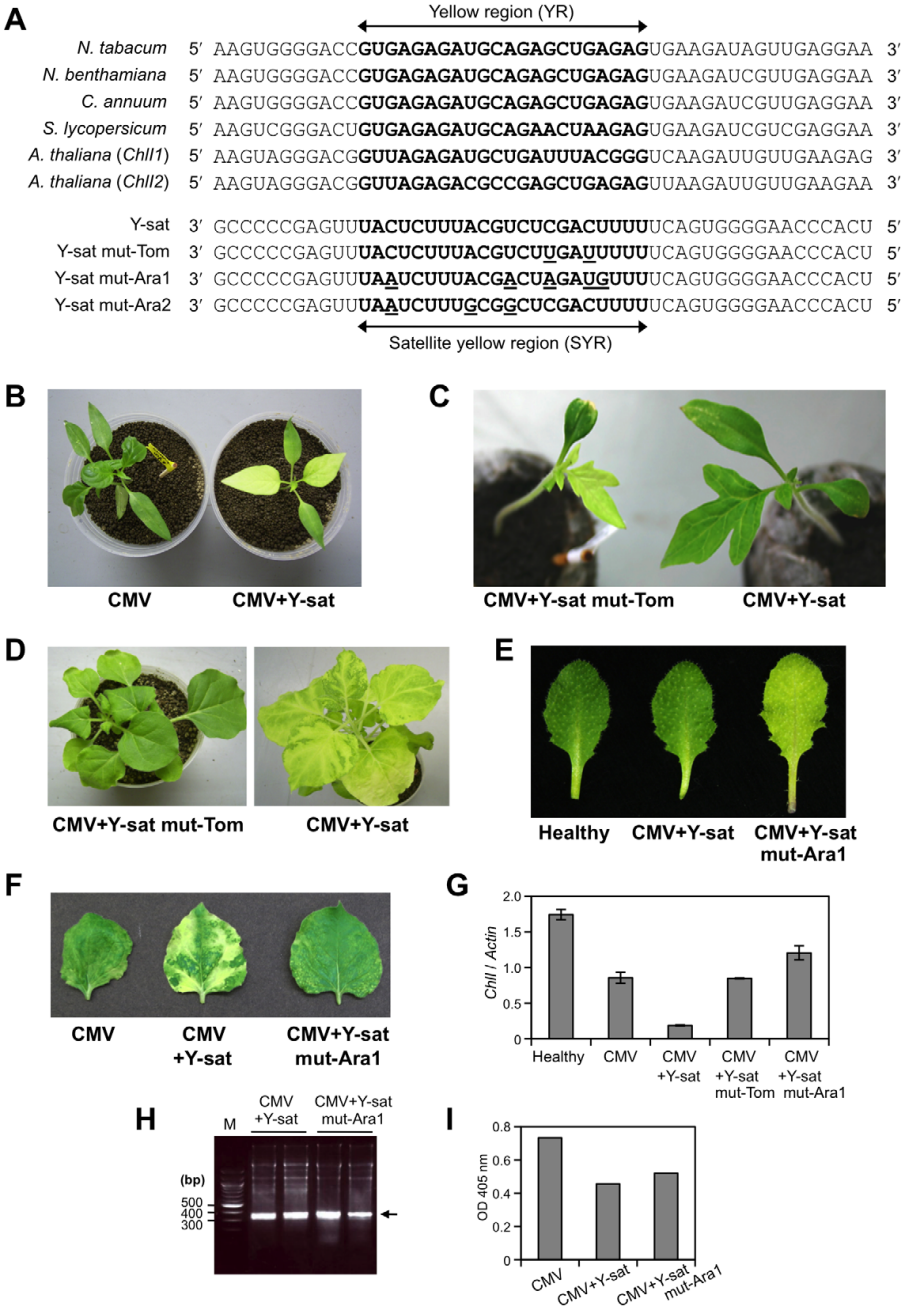
Sequence complementarity between Y-sat and the host *ChlI* gene is important for the induction of yellowing. A. The 22-nt complementary sequences of the *ChlI* genes of various plants and Y-sat or Y-sat mutants. The 22-nt complementary sequences in the *ChlI* genes (yellow region; YR) and in the Y-sat (satellite yellow region; SYR) are in bold face. Site-directed mutations were introduced in the SYR of Y-sat so that the generated Y-sat mutants (Y-sat mut-Tom, Y-sat mut-Ara1 and Y-sat mut-Ara2) have the 22-nt complementary sequence including G-U pairs to the host YR in *ChlI* of tomato, *Arabidopsis ChlI1* and *ChlI2*, respectively. Introduced mutations of the Y-sat mutants are underlined. B. Yellow phenotype of pepper infected with CMV-Y (left) or CMV-Y and Y-sat (right) at 10 dpi. Pepper has the same YR sequence in the *ChlI* gene as that of tobacco. C. Yellow phenotype of tomato infected with CMV-Y and Y-sat mutant, Y-sat mut-Tom at 10 dpi. Left, tomato plant infected with CMV-Y and Y-sat mut-Tom; right, tomato plant infected with CMV-Y and Y-sat. D. Green mosaic on *N*. *benthamiana* infected with CMV-Y and Y-sat mut-Tom at 14 dpi. Left, *N*. *benthamiana* infected with CMV-Y and Y-sat mut-Tom; right, *N*. *benthamiana* infected with CMV-Y and Y-sat. E. Yellow phenotype of *Arabidopsis* infected with CMV-Y and Y-sat mut-Ara1 at 10 dpi. F. Green mosaic symptoms on *N*. *benthamiana* infected with CMV-Y and Y-sat mut-Ara1 at 14 dpi. Left, *N*. *benthamiana* infected with CMV-Y; middle, *N*. *benthamiana* infected with CMV-Y and Y-sat; right, *N*. *benthamiana* infected with CMV-Y and Y-sat mut-Ara1. G. The mRNA levels of *ChlI* in Y-sat mutant-infected *N*. *benthamiana* determined by quantitative real-time RT-PCR. RNAs were extracted from equivalent systemic leaves infected with CMV, CMV+Y-sat or CMV+Y-sat mutant, and equivalent leaves of healthy *N. benthamiana*. *ChlI* mRNA levels relative to the *actin* mRNA level are shown (mean ± SE; *n* = 3). H. SatRNAs accumulation in Y-sat- and Y-sat mut-Ara1-infected *N. benthamiana*. Total RNAs were extracted from the *N. benthamiana* leaves shown in panel F. An ethidium bromide-stained 1.2% agarose gel is shown. I. CMV accumulation at 14 dpi in systemic leaves of *N*. *benthamiana* inoculated with CMV-Y and Y-sat mut-Ara1, which was determined by conventional ELISA using antibodies raised against CMV CP. Samples are those shown in panel F.

The *ChlI* genes of pepper, tomato and *Arabidopsis thaliana* were obtained from the gene database, and the 22-nt complementary sequences of the *ChlI* genes and Y-sat were aligned (Figure 4A). Pepper has the same YR sequence in the *ChlI* gene as those of tobacco and *N*. *benthamiana*. Conversely, several mismatches were found in the case of the tomato *ChlI* and *Arabidopsis ChlI* (*ChlI1* and *ChlI2*) genes (Figure 4A). We next examined whether the Y-sat can induce yellow symptoms on pepper, tomato and *Arabidopsis* plants. As expected, infected pepper plants developed bright yellow symptoms (Figure 4B, right plant), whereas tomato plants did not (Figure 4C, right plant). By site-directed mutagenesis of the SYR, we generated three Y-sat derivatives having the 22-nt continuous sequence complementary to the corresponding YRs of tomato *ChlI* gene, *Arabidopsis ChlI1* and *ChlI2* genes (Y-sat-Tom, Y-sat-Ara1 and Y-sat-Ara2, respectively) (Figure 4A). When tomato plants were inoculated with the Y-sat mut-Tom and the helper virus, yellow symptoms appeared at 10 dpi (Figure 4C, left plant). However, some of the introduced mutations in individual plants had reverted to the original nucleotides at 21 dpi. Notably, the Y-sat mut-Tom did not induce yellow symptoms in *N*. *benthamiana* (Figure 4D, left plant). Similarly, when *Arabidopsis* plants were infected with CMV-Y and Y-sat mut-Ara1, yellow symptoms appeared (Figure 4E, right plant). On the other hand, Y-sat mut-Ara2 did not induce yellowing (data not shown). The last observation is consistent with the previous studies by Huang and Li [32], who reported that *ChlI2* of *Arabidopsis* has lower functionality than *ChlI1* due to a reduced level of expression. In addition, like Y-sat mut-Tom, Y-sat mut-Ara1 did not induce yellowing in *N*. *benthamiana* (Figure 4F). Quantitative real-time RT-PCRs confirmed that the mRNA levels of the *ChlI* gene in the Y-sat mutants-infected *N. benthamiana* plants were not downregulated, unlike in the Y-sat-infected plant (Figure 4G). There were little differences in satRNA or viral accumulation between Y-sat-infected- and Y-sat mut-Ara1-infected leaves of *N*. *benthamiana* (Figure 4H and 4I), confirming that the Y-sat mutant was replicated to a level similar to that of the original Y-sat in the systemic leaves of *N. benthamiana*. These results, all together, strongly suggest that a specific interaction between Y-sat and the *ChlI* host gene is involved in development of the yellow symptom.

### Massive amounts of small RNAs from SYR sequence accumulate in Y-sat-infected plants

**Figure 5:**
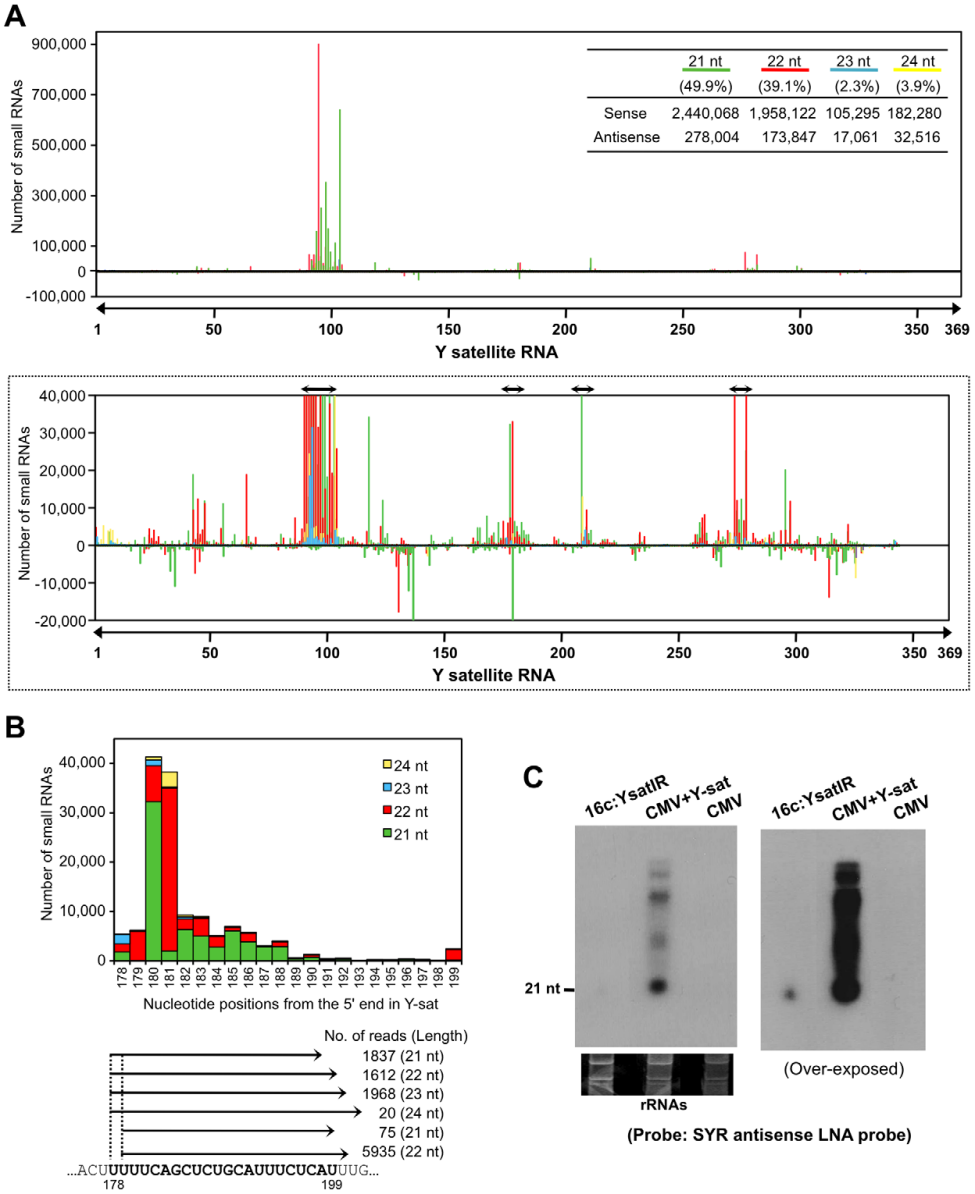
Small RNAs generated from SYR in Y-sat sequence. A. Deep-sequencing analysis of the Y-sat small RNAs in *N*. *benthamiana* plants infected with CMV-Y and Y-sat. Location and frequency of Y-sat-derived small RNAs (21–24 nt) were mapped to the Y-sat sequence in either sense (above the *x*-axis) or antisense (below the *x*-axis) orientation. Data from 21-, 22-, 23-, and 24-nt small RNAs are color-coded in green (21 nt), red (22 nt), blue (23 nt) and yellow (24 nt). The table shows the small RNA reads and percentage of each size of the small RNA. The graph surrounded by broken lines shows an enlarged graph covering the number of small RNAs from –20,000 to 40,000 for better visibility of the small RNAs distribution. Double-headed arrows show the major hot spots where abundant siRNAs are generated from the Y-sat. B. Histogram of location, frequency and size distribution of small RNAs corresponding to the satellite yellow region (SYR) in Y-sat. Numbers on *x*-axis refer to location of SYR in the Y-sat sequence. The number of reads for the siRNAs homologous to SYR is given below the histogram. SYR is in bold face. C. Detection of siRNAs corresponding to SYR in 16c:YsatIR and Y-sat-infected plans in Northern blots. As a helper virus, CMV strain Y was used. LNA probes specific to SYR of Y-sat were used for hybridization. Ribosomal RNAs were used as a loading control. Left image was taken after 4 h exposure; right image is an overnight exposure of the same membrane.

Because Y-sat and the host *ChlI* gene seemed to have a specific interaction through their sequence complementarity, we then examined the possible involvement of RNA silencing in the Y-sat-mediated yellow phenotype. First, we tested whether the Y-sat-derived siRNAs can be hybridized and detected by *ChlI* mRNA probe. As shown in Figure S2, sense siRNAs from Y-sat in both Y-sat-infected and 16c:YsatIR plants were clearly detected in Northern blots using the *ChlI* sense RNA probe. On the other hand, we failed to detect antisense siRNAs from Y-sat by Northern blots using the *ChlI* antisense RNA probe. This result seems reasonable because the YR and SYR sequences do not share complementarity in the antisense orientation (Figure S2). In addition, we also detected siRNAs derived from Y-sat mut-Ara1 using the *Arabidopsis ChlI1* sense RNA in Northern blots. As shown in Figure S3, 351-bp *Arabidopsis ChlI1* sense RNA probe, which contains the 22-nt sequence complementary to Y-sat mut-Ara1, detected the siRNAs of Y-sat mut-Ara1 in the *Arabidopsis* leaves infected with CMV and Y-sat mut-Ara1. Assuming that the yellow symptoms are the result of post-transcriptional RNA silencing of host genes directed by Y-sat specific sequences, we further analyzed Y-sat-derived siRNAs profile to find whether Y-sat siRNAs targeting the *ChlI* mRNA accumulate in the Y-sat-infected plants. We thus conducted small RNA deep sequencing to map the small RNAs on the Y-sat sequence. As the result, Y-sat-derived siRNAs covered almost the entire Y-sat sequence, and the majority of Y-sat siRNAs accumulated in the sense orientation in the Y-sat-infected plants. In addition, 21-nt and 22-nt siRNAs were abundant among the Y-sat small RNAs populations (Figure 5A). Y-sat-derived siRNAs in both sense and antisense orientation were non-uniformly distributed along the sequence with a few small RNA-generating hot spots (Figure 5A). Abundant siRNAs were accumulated from the regions around positions 100, 180, 211 and 280 on the Y-sat. Northern hybridization confirmed that the most abundant siRNAs were generated from the region at positions 1–200 as opposed to 201–369 (Figure S4). Furthermore, we found abundant siRNAs homologous to the SYR (Figure 5B). The accumulation of siRNAs corresponding to SYR in 16c:YsatIR and Ysat-infected plans was confirmed by Northern hybridization using LNA probes specific to SYR of Y-sat (Figure 5C). In deep-sequencing analysis, we also identified the *ChlI* siRNAs in the Y-sat-infected tissues although the amounts were not very high (Figure S5). The profile of the *ChlI* siRNAs revealed a very unique feature; all siRNAs derived from *ChlI* were generated only from the 3′ region downstream of the cleavage site as described below.

### Small RNAs from Y-sat SYR cleave *ChlI* mRNA post-transcriptionally

**Figure 6:**
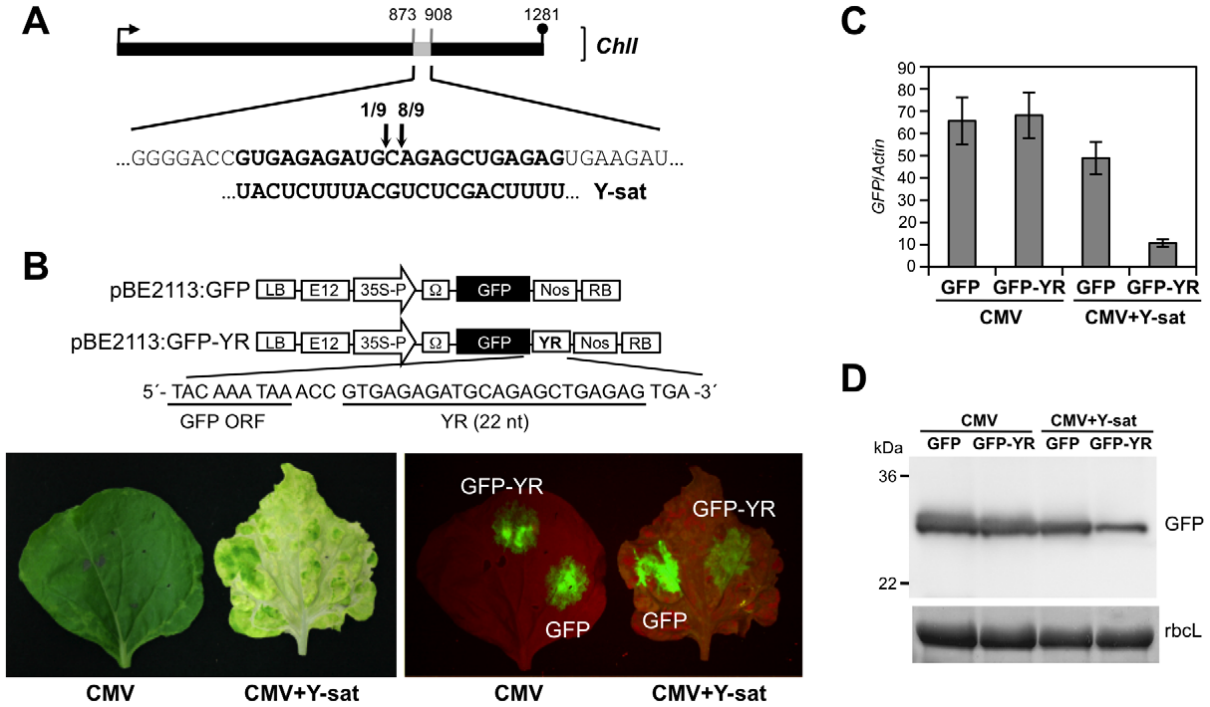
Sequence complementarity between Y-sat and the *ChlI* gene triggers mRNA cleavage, causing a yellow symptom. A. Schematic illustration of the *ChlI* gene derived from the 5′-end analyses of the cleaved *ChlI* mRNAs. The number of 5′-RACE clones corresponding to each site is indicated by arrows. B. Y-sat-mediated targeting of GFP-YR sensor mRNAs. Schematic representation of the GFP-YR sensor construct is shown above. Leaf images show GFP fluorescence expressed from the GFP-YR sensor construct by agroinfiltration in *N. benthamiana* leaves with or without Y-sat infection. As a helper virus, CMV strain Y was used. The GFP fluorescence in the infiltrated patches was observed 2 days after agroinfiltration. Note that the GFP fluorescence (GFP-YR) was reduced in Y-sat-infected leaves, suggesting that YR was specifically targeted by Y-sat. C. The mRNA levels of *GFP* determined by quantitative real-time RT-PCR. Total RNAs were extracted from agroinfiltrated areas at 3 days after agroinfiltration. The mRNA levels for *actin* were used for data normalization. D. Western blot analysis of GFP. Total proteins were extracted from agroinfiltrated areas at 3 days after infiltration and subjected to western blot analysis using anti-GFP antibodies. RuBisCo large subunit (rbcL) stained with Coomassie brilliant blue (CBB) is shown as a loading control.

To clarify whether the *ChlI* mRNA is cleaved in the Y-sat-infected plant, we analyzed the 5′ ends of the cleaved mRNA products with a 5′-RACE assay. Sequencing of the 5′-RACE products revealed two distinct cleavage sites in the YR of the *ChlI* mRNA. Almost all identified cleavage sites were mapped at the middle position in YR (between 890 and 891), which agrees with the expected cleavage site(s) driven by the 21-nt and 22-nt siRNAs (Figure 6A). To verify that Y-sat can direct sequence-specific cleavage, we created a GFP sensor construct in which the 3′ non-coding region contained the 22-nt YR sequence (Figure 6B). The construct was delivered by agroinfiltration into Y-sat-infected *N. benthamiana* leaves that had bright yellow symptoms (Figure 6B). GFP accumulation was monitored using UV light after agroinfiltration. As shown in Figure 6B, GFP fluorescence was reduced in the Y-sat-infected tissues, and this observation was supported by the results of quantitative real-time RT-PCR of the *GFP* mRNA (Figure 6C). The accumulation of GFP protein was also reduced in the Y-sat-infected tissues (Figure 6D). These results clearly demonstrated that the 22-nt YR sequence in the sensor mRNA was sufficient for the sequence-specific downregulation of GFP-YR mRNA in Y-sat-infected tissues.

## Discussion

Plant RNA silencing has often been implicated as a molecular mechanism for symptom induction caused by viruses or viral subviral agents. Viral suppressors of RNA silencing (VSRs) are able to compromise the endogenous RNA silencing pathways [19], [20], and these virus-encoded silencing suppressors have also been identified as pathogenicity determinants. Indeed, virus-induced developmental abnormalities are often explained by the interference of virus-encoded VSRs with host miRNAs involved in the developmental processes [39], [40]. However, no explanation for specific symptoms caused by VSRs has ever been confirmed nor has any report explained the molecular basis for a specific viral symptom including yellowing and necrosis. In recent studies, host mRNAs were identified as potential targets of siRNAs and miRNAs in virus-infected tissues, and several have been proved to be downregulated [25], [26]. For example, Moissiard and Voinnet [25] demonstrated that the *RCC1* gene in *Arabidopsis* infected with *Cauliflower mosaic virus* (CaMV) was downregulated by virus-derived siRNAs, but contrary to expectations, the decrease in gene expression did not affect either viral accumulation or symptoms. It is, in fact, quite difficult to clarify the relationship between such small RNAs and viral pathogenicity although the idea that host gene silencing against a particular gene might contribute to the specific expression of symptoms is very attractive.

**Figure 7:**
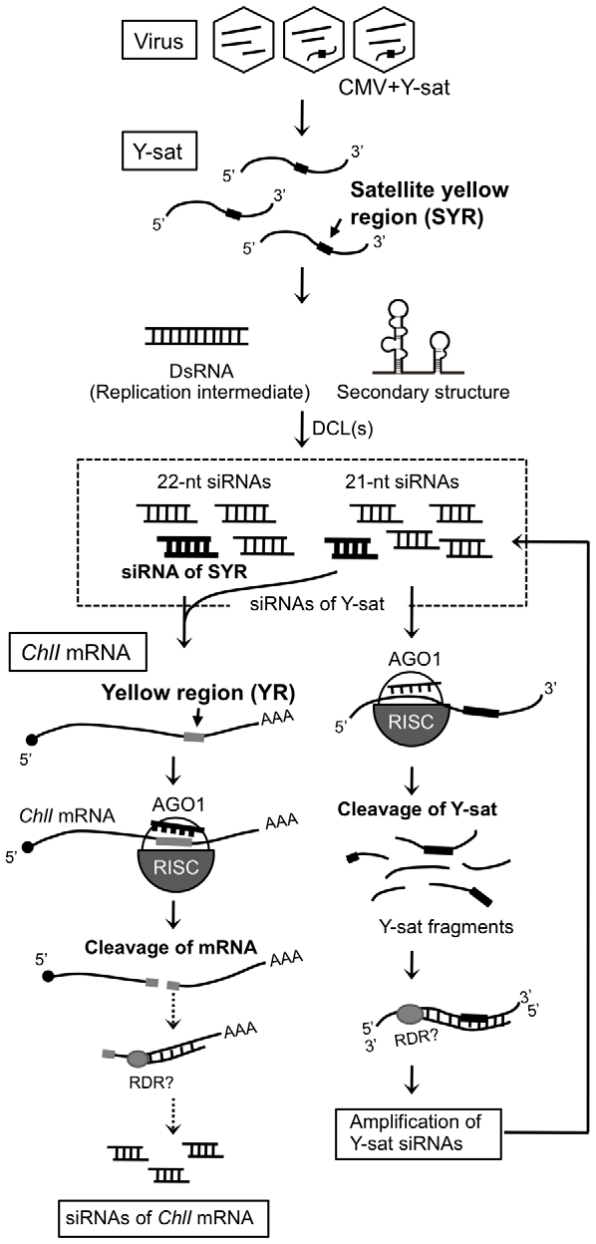
Scenarios to explain the cleavage of *ChlI* mRNA by Y-sat in the RNA silencing pathway. The most probable scenario is that AGO1 associated with the primary Y-sat siRNAs cleaves the *ChlI* mRNA at the SYR-YR portion, then a host RNA-dependent RNA polymerase (RDR) may access the cleaved *ChlI* mRNA fragments to produce dsRNAs, which are subsequently processed into secondary siRNAs. In addition, Y-sat itself is cleaved by AGO1 loaded with siRNAs derived from Y-sat, inducing the production of a large amount of siRNAs containing the SYR sequence.

In the present study, we have shown that siRNAs derived from Y-sat induced bright yellow mosaics on tobacco by specifically targeting mRNA of the host *ChlI* gene, resulting in the inhibition of chlorophyll biosynthesis. Here we provide several lines of evidence that Y-sat-induced bright yellow mosaics are the outcome of specific interference between the pathogen-derived siRNAs and a host gene. First, the 22-nt long region of Y-sat (SYR) produces specific siRNAs that were complementary, including four G-U pairs, to the 22-nt long region of tobacco *ChlI* mRNA (YR). Second, the *ChlI* mRNA could detect Y-sat-derived siRNAs in Northern blots. Third, 5′-RACE experiments revealed that the *ChlI* mRNA was cleaved exactly in the expected middle of the YR. Fourth, the levels of the *ChlI* transcript significantly decreased in both Y-sat-infected plants and the transgenic plants expressing Y-sat dsRNA. Fifth, the Y-sat mutants that had the modified SYR to either *Arabidopsis ChlI1* mRNA or tomato *ChlI* mRNA were able to induce yellow symptoms in these host plants. In contrast, these modified Y-sat lost the ability to induce yellow symptoms on tobacco. Sixth, the GFP sensor construct carrying the YR sequence was specifically targeted in Y-sat-infected plants. Considering all these results, we propose a model that explains that the Y-sat-mediated yellow symptom results from the cleavage of host *ChlI* mRNA by RNA silencing machinery (Figure 7).

In deep-sequencing analysis, we found abundant Y-sat-derived siRNAs in the Y-sat-infected *N. benthamiana*. Furthermore, we noticed that the *ChlI*-derived siRNAs also accumulated in the Y-sat-infected tissues although the amounts were not very high. The profile of the *ChlI* siRNAs was very unique because all siRNAs derived from *ChlI* were generated only from the 3′ region downstream of the cleavage site (Figure S5). Importantly, spread of RNA silencing beyond the targeting site in endogenous plant genes has not been shown [30], [41], [42], except for *trans*-acting siRNAs [43]. Whether secondary siRNAs can be generated from the *ChlI* mRNA after vsiRNA-directed cleavage, and whether such secondary siRNAs are involved in the downregulation of the *ChlI* gene still need careful studies.

Here we propose that Y-sat caused the yellow symptoms on tobacco by directing post-transcriptional RNA silencing against the *ChlI* mRNA. However, yellow symptoms appeared much brighter in Y-sat-infected plants than in 16c:YsatIR plants (Figure 1B). With regard to the observation, the amount of Y-sat-derived siRNAs in 16c:YsatIR plants was lower than in Y-sat-infected plants (Figure 5C), probably leading to different yellow phenotype between 16c:YsatIR plants and Y-sat-infected plants. Indeed, the level of the *ChlI* transcript analyzed by the Northern blot was higher in the 16c:YsatIR plants than in the Y-sat-infected plants (Figure 2B). Alternatively, as suggested by Du et al. [44], Y-sat siRNAs from secondary structures (T-shaped hairpins) may predominate over the Y-sat siRNAs generated from perfect dsRNA forms. Thus it is likely that RNA silencing against *ChlI* and subsequent yellow phenotype can vary depending on the qualities and amounts of siRNAs derived from satRNA.

In conclusion, we discovered the molecular basis of the symptom modifications induced by Y-sat: the involvement of RNA silencing mechanism in the pathogenicity of Y-sat. But the molecular mechanism underlying the synergistic and/or antagonistic interaction between satRNAs, helper viruses and host plants still remain to be explored. In addition, the origin(s) of satRNAs, their evolutionary strategy and biological significance have long been intriguing topics. Since the original isolation of Y-sat in Japan more than 30 years ago [4], no other satRNAs that induce yellow mosaics on tobacco have been isolated in the world, suggesting that Y-sat is a rare satRNA that specifically induces yellow mosaics on tobacco. We have observed that Y-sat cannot compete with other similar size satRNAs [35], and thus Y-sat may survive through a different strategy from other satRNAs; the Y-sat-induced yellowing of leaves, which could preferentially attract aphids (the vectors of CMV and its satRNAs), may have favored the transfer of CMV that harbors Y-sat during the its evolutionary history.

## Materials and Methods

### Plant materials

*Nicotiana benthamiana*, *Capsicum annuum*, *Solanum lycopersicum* and *Arabidopsis thaliana* were used as host plants for the analysis. *Nicotiana benthamiana* line 16c having a single copy of the GFP transgene [45] was obtained from Dr. D. Baulcombe (Sainsbury Laboratory, UK) and was also used for the analysis. All plants were grown in a plant growth room with a 16-h light/8-h dark at 24°C and 50% relative humidity.

Transgenic *N. benthamiana* lines expressing the inverted repeat (IR) of Y-sat were generated by transforming *N. benthamiana* 16c with the binary vector pIG121-Hm carrying the IR of Y-sat under the CaMV 35S promoter. In the sense and antisense orientations, the 317-bp (53 to 369) Y-sat sequence (GenBank accession D00542) was inserted in the pJM007 vector [46], then the inverted repeat (IR)-expressing cassette was transferred to a Ti-plasmid vector, pIG121-Hm. The Ti plasmid vector containing the IR (1004 nt) of the GUS sequence (GUS-IR) was previously constructed [47].

### Virus materials and inoculation

CMV strain Y (CMV-Y) was used as a helper virus for satellite RNA. To induce gene silencing to the *ChlI* gene, we used two CMV-based vectors, CMV-A1 and CMV-H1. CMV-A1 and CMV-H1 are derived from RNA2 of CMV-Y, and CMV-A1 lacks the C-terminal one-third of the intact 2b protein as a consequence of introducing a multiple cloning site [37], while CMV-H1 vector lacks the entire 2b protein [38]. The 150-bp of the *ChlI* gene (817 to 966) was inserted into the CMV vectors to create CMV-A1:ChlI150 and CMV-H1:ChlI150, respectively. To avoid severe mosaic symptom induction by CMV-Y, we used a pseudorecombinant virus that contains RNA components derived from RNA1 and RNA3 of CMV strain L together with RNA2 of the vector. Each plasmid containing a full-length cDNA clone of RNA1 to RNA3 was transcribed in vitro after linearization with a restriction enzyme [37]. Infectious viruses were then created by mixing transcripts of RNAs 1 to 3. For virus propagation, leaves of 4-week-old plants of *N*. *benthamiana* were dusted with carborundum and rub-inoculated with the RNA transcripts. For inoculation of tomato plants, leaves of young plants were rub-inoculated with the sap from virus-infected tissues of *N*. *benthamiana*. Successful systemic infection with the virus containing the full insert sequence was confirmed by RT-PCRs. Viral accumulation was examined by conventional ELISA [48] using the antibodies raised against the CMV CP.

### RNA analyses

Total RNAs were extracted by either a conventional phenol/chloroform method [47] or a method using Trizol reagent (Invitrogen) following the manufacturer's instructions. The *N*. *benthamiana ChlI* clone including the entire ORF was amplified by RT-PCR using the primer pair designed from the tobacco *ChlI* sequence (5′-GCTCTAGAATGGCTTCACTACTAGGAAC-3′ for forward primer, 5′-GCCCAAGCTTAGGCGAAAACCTCATAAAATTTC-3′ for reverse primer). Quantitative real-time RT-PCR was performed essentially as described before [37]. Primers for quantitative real-time RT-PCR for the *N*. *benthamiana ChlI* gene were as follows: 5′-CTTATTGGTTCGGGTAATCCTG-3′ for forward primer and 5′-GCTGAGTCGATTTGGTTCTG-3′ for reverse primer. The *N. benthamiana actin* gene was amplified using 5′-GCGGGAAATTGTTAGGGATGT-3′ for forward primer and 5′-CCATCAGGCAGCTCGTAGCT-3′ for reverse primer and used for data normalization. Northern blot hybridization was performed essentially as previously described [49]. Specific probe for the *ChlI* gene was generated by PCR with the PCR DIG Probe Synthesis Kit (Roche Diagnostics) to amplify the 371 bp (634 to 1004) of 3′-terminal regions of the *ChlI* gene using the primer pair ChlI-634F (5′-GAGCCTGGTCTTCTTGCTAAAGC-3′) and ChlI-1004R (5′-GCTGAGTCGATTTGGTTCTG-3′). In the Northern blots of the small RNAs corresponding to the 22-nt complementary sequence region (satellite yellow region, SYR), the SYRs were detected by using ^32^P-labeled locked nucleic acid (LNA) oligonucleotide probes described previously [50].

The *ChlI* mRNA cleavage sites were analyzed by modified RNA-ligase mediated 5′-RACE [51]. Total RNA (10 µg) was purified using the MicroPoly(A) Purist Kit (Ambion), then the fractionated Poly(A)^+^ mRNA was ligated to the GeneRacer RNA Oligo adaptor using the GeneRacer Kit (Invitrogen). Ligated RNAs were reverse transcribed using the gene-specific reverse primer for the *ChlI* gene, ChlI-1004R (5′-GCTGAGTCGATTTGGTTCTG-3′). The 5′end of the cDNA was then amplified by PCR using the GeneRacer 5′ primer and the gene-specific reverse primer used for the reverse transcription for the first PCR. The GeneRacer 5′ nested primer was also used for the subsequent nested PCR. The amplified product from the nested PCR was excised from 1.2% agarose gel and cloned into pGEM-T Easy (Promega) for sequencing.

### Protoplast experiments

Protoplasts were prepared from leaves of *N*. *benthamiana* as described before [52]. The dsRNA of four satRNAs (Y-sat, S19-sat and T73-sat [35] and CM-sat [36]) were used. DsRNA of satRNA was prepared by in vitro transcription using a PCR-amplified fragment containing the T7 promoter sequence as described previously [52]. The prepared protoplasts were transfected with the satRNA dsRNAs (2 µg) in a PEG–calcium solution as described [52] and then incubated for 20 h. Total RNA was extracted from the harvested protoplasts with Trizol reagent (Invitrogen), and the mRNA levels of the *ChlI* and *CAB* gene were measured by quantitative real-time RT-PCR (mean ± SE; *n* = 3). Primers for quantitative real-time RT-PCR for the *CAB* gene were 5′-CGGCCGATCCAGAAACTTT-3′ for forward primer and 5′-GCCCATCTGCAGTGAATAACC-3′ for reverse primer.

### Deep-sequencing analysis

Total RNA was extracted from CMV and Y-sat-infected *N. benthamiana* plants. Small RNAs were isolated essentially as described [49] and submitted to Hokkaido System Science (Sapporo, Japan), where deep-sequencing analysis was performed on an Illumina Genome Analyzer using the standard protocol of the manufacturer. The 18–45-nt small RNA reads were extracted from raw reads and aligned with the Y-sat sequence using the program SOAP [53] to search for perfectly matched sequences.

### GFP sensor experiments

The GFP-YR sensor gene was inserted between the *Bam*HI and *Sac*I sites in the pBE2113 vector. The Ti-plasmid construct was then introduced into *Agrobacterium tumefaciens* KYRT1 strain, which was supplied by Dr. G. B. Collins (University of Kentucky, USA). Agrobacterium infiltration was carried out essentially as described [49].

### Western blot analysis

Total proteins were extracted from the sample tissues by grinding in Laemmli buffer, separated by SDS-PAGE, and /transferred onto a PVDF membrane (Immobilon, Millipore). Anti-GFP antibodies were purchased from Roche and used at a 1∶1000 dilution. For immunostaining, an alkaline phosphatase-conjugated goat anti-rabbit antibody was added to the blots at a 1∶3000 dilution followed by colorimetric development with BCIP and NBT.

## Supporting Information

Figure S1 Two-dimensional electrophoresis of extracted proteins from *Nicotiana benthamiana* 16c (16c, upper panel) and *N*. *benthamiana* 16c:YsatIR (16c:YsatIR, lower panel). Red circles in the gel of 16c indicate the spots that decreased in 16c:YsatIR compared to 16c. Blue circles in the gel of 16c:YsatIR indicate the spots that increased in 16c:YsatIR compared to 16c. Among these spots, we selected five spots (M1-M5) that had markedly changed between 16c and 16c:YsatIR for LC-MSMS analysis. The analyzed proteins were identified as follows: M1, ribulose bisphosphate carboxylase large chain (RuBisCo large subunit); M2, ribulose bisphosphate carboxylase activase; M3, glyceraldehyde-3-phosphate dehydrogenase A (NADP-dependent glyceraldehydephosphate dehydrogenase subunit A); M4, ribulose bisphosphate carboxylase small chain 1 (RuBisCo small subunit 1); M5, ribulose bisphosphate carboxylase small chain 1 (RuBisCo small subunit 1). This proteome analysis revealed that chloroplast-related proteins were significantly altered in 16c:YsatIR, and that the mobility of the RuBisCo small subunit had shifted in a two-dimensional gel, suggesting that RuBisCo small subunit in 16c:YsatIR was modified at the posttranslational level.
(TIF)

Figure S2 Detection of Y-sat small RNAs by the *ChlI* gene probe in Northern blots. RNAs were prepared from 16c:YsatIR, 16c and CMV-infected *N*. *benthamiana* with or without Y-sat. Left panel, detection of sense small RNAs of Y-sat by the hybridization with the *ChlI* sense RNA (mRNA) probe. Right panel, detection of antisense small RNAs of Y-sat by the hybridization with the *ChlI* antisense RNA probe. For the RNA probe, the amplified *ChlI* fragments (634–1004, 371bp) were cloned downstream of the T7 promoter in the pGEM-T easy vector (Promega). The sense and antisense RNA probes specific to the *ChlI* were prepared using DIG RNA Labeling Mix (Roche). Arrows indicate small RNAs of Y-sat. The 22-nt sequence complementarity between the *ChlI* and Y-sat is shown below each panel. A continuous 22-nt complementary sequence including G-U pairs is formed between the *ChlI* sense RNA and Y-sat sense RNA, but there are four mismatches in the region between the *ChlI* antisense RNA and Y-sat antisense RNA.
(TIF)

Figure S3 Northern blots of Y-sat mut-Ara1 small RNAs in Y-sat mut-Ara1-infected *Arabidopsis*. RNAs were prepared from *Arabidopsis* leaves infected with CMV or CMV+Y-sat mut-Ara1. For the RNA probe, the amplified *ChlI1* fragments (750–1100, 351bp) were cloned downstream of the T7 promoter in the pGEM-T easy vector (Promega). The sense RNA probe specific to the *Arabidopsis ChlI1* was prepared using DIG RNA Labeling Mix (Roche). Note that *Arabidopsis ChlI1* sense RNA probe detected small RNAs from Y-sat mut-Ara1 (shown by an arrow) in the lane for Y-sat mut-Ara1-infected leaves.
(TIF)

Figure S4 Confirmation of the relative abundance of Y-sat small RNAs by Northern blot hybridization. The small RNAs derived from the hot spots that were observed in the Y-sat small RNA profiles (Figure 5A) were detected and validated using DIG-labeled probes: Y-sat-1-200 and Y-sat-201-369. Y-sat-1-200 is complementary to the positions 1–200, and Y-sat-201-369 is complementary to the positions 201–369. The hybridization signals detected by Y-sat-1-200 (shown by an arrow in the left panel) were clearly stronger than those detected by Y-sat-201-369 (shown by an arrow in the right panel). These results support that the deep-sequencing approach reflects the hot spots identified for Y-sat small RNAs.
(TIF)

Figure S5 Deep-sequencing analysis of the *ChlI* small RNAs in *N*. *benthamiana* plants infected with CMV-Y and Y-sat. Location and frequency of the *ChlI*-derived small RNAs (21- to 24-nt) were mapped to the *ChlI* sequence in either sense (above the *x*-axis) or antisense (below the *x*-axis) orientation. Data from 21-, 22-, 24-nt small RNA are color-coded in green (21 nt), red (22 nt), and yellow (24 nt). Table in graph gives the number of small RNA reads and percentage of each size. Note that the small RNAs are mostly generated from the 3′ region downstream of the cleavage site indicated by an arrow.
(TIF)

Table S1 Genes downregulated in 16c:YsatIR 40% less than in 16c plants in microarray analysis. Among the 134 genes, 31 genes were chloroplast-related genes.
(DOC)

Text S1 Supplementary materials and methods for the DNA microarray (Table S1) and two-dimensional electrophoresis experiments (Figure S1).
(DOC)
