# Supplementary figures and images for: A Viral Satellite RNA Induces Yellow Symptoms on Tobacco by Targeting a Gene Involved in Chlorophyll Biosynthesis using the RNA Silencing Machinery
Source: PLoS Pathog. 2011 May 5;7(5):e1002021. doi: 10.1371/journal.ppat.1002021 (PMC3088725; doi:10.1371/journal.ppat.1002021)

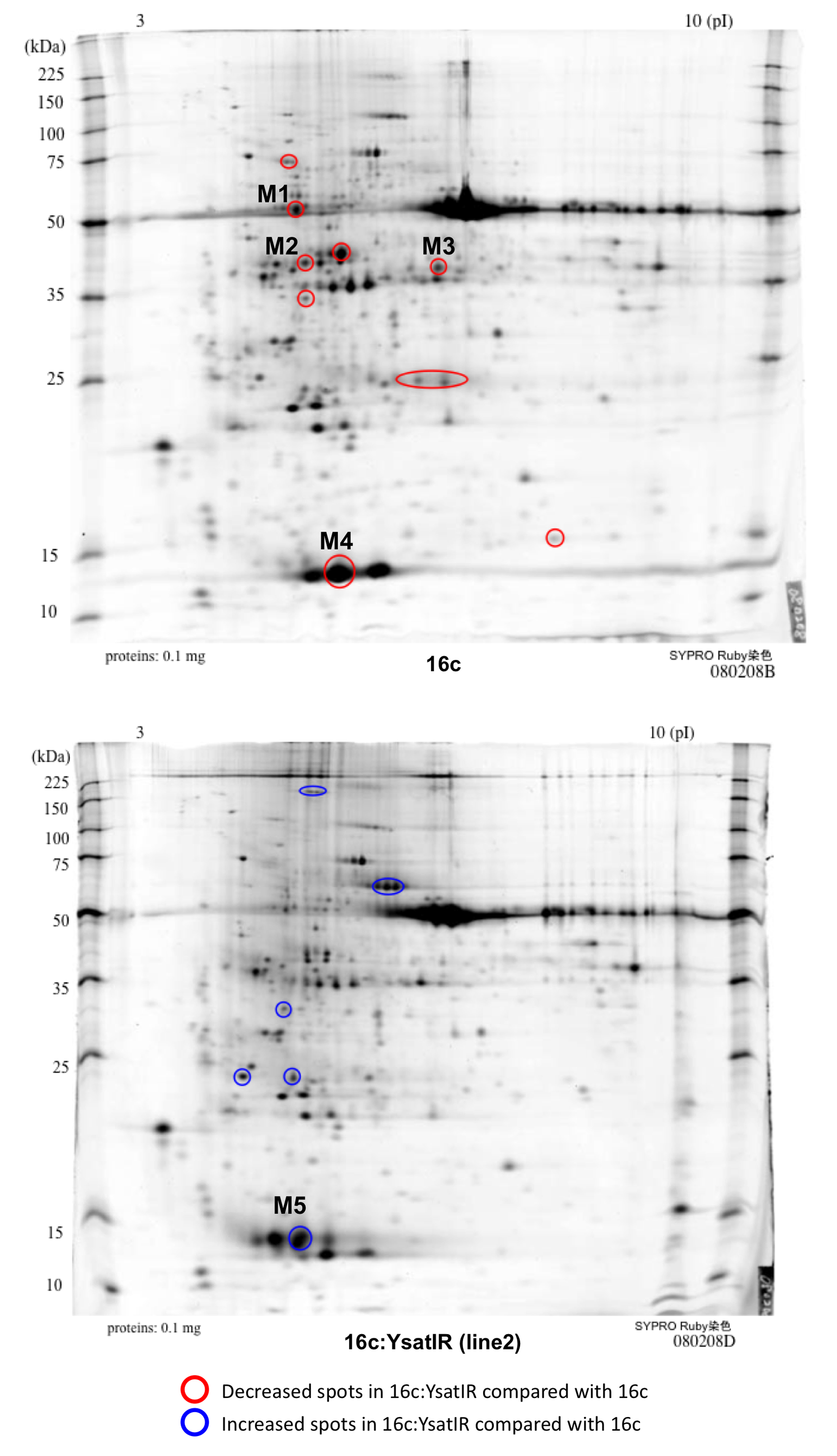

Supplement: Figure S1 — Two-dimensional electrophoresis of extracted proteins from Nicotiana benthamiana 16c (16c, upper panel) and N. benthamiana 16c:YsatIR (16c:YsatIR, lower panel). Red circles in the gel of 16c indicate the spots that decreased in 16c:YsatIR compared to 16c. Blue circles in the gel of 16c:YsatIR indicate the spots that increased in 16c:YsatIR compared to 16c. Among these spots, we selected five spots (M1-M5) that had markedly changed between 16c and 16c:YsatIR for LC-MSMS analysis. The analyzed proteins were identified as follows: M1, ribulose bisphosphate carboxylase large chain (RuBisCo large subunit); M2, ribulose bisphosphate carboxylase activase; M3, glyceraldehyde-3-phosphate dehydrogenase A (NADP-dependent glyceraldehydephosphate dehydrogenase subunit A); M4, ribulose bisphosphate carboxylase small chain 1 (RuBisCo small subunit 1); M5, ribulose bisphosphate carboxylase small chain 1 (RuBisCo small subunit 1). This proteome analysis revealed that chloroplast-related proteins were significantly altered in 16c:YsatIR, and that the mobility of the RuBisCo small subunit had shifted in a two-dimensional gel, suggesting that RuBisCo small subunit in 16c:YsatIR was modified at the posttranslational level. (TIF) [file ppat.1002021.s001.tif]

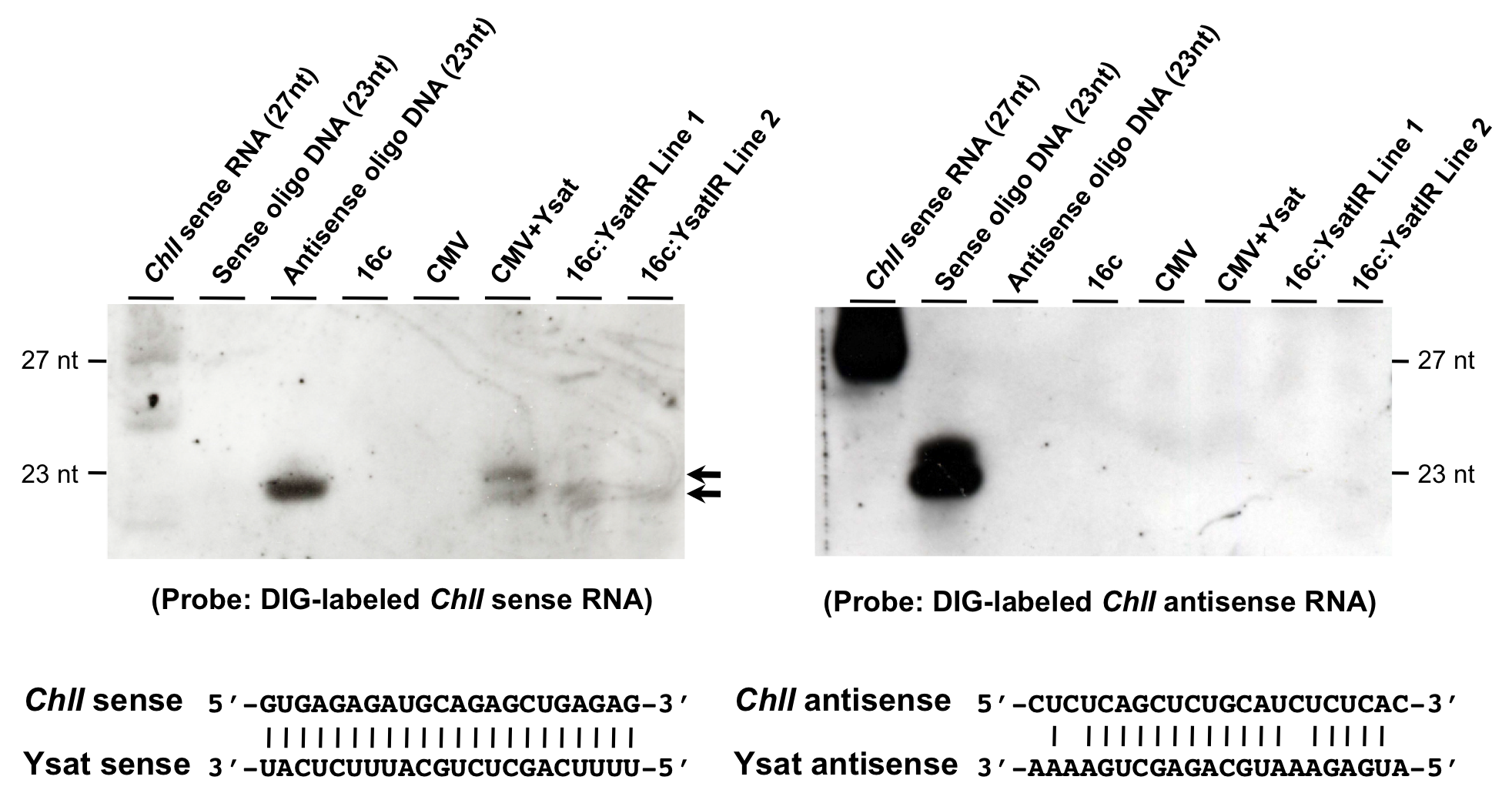

Supplement: Figure S2 — Detection of Y-sat small RNAs by the ChlI gene probe in Northern blots. RNAs were prepared from 16c:YsatIR, 16c and CMV-infected N. benthamiana with or without Y-sat. Left panel, detection of sense small RNAs of Y-sat by the hybridization with the ChlI sense RNA (mRNA) probe. Right panel, detection of antisense small RNAs of Y-sat by the hybridization with the ChlI antisense RNA probe. For the RNA probe, the amplified ChlI fragments (634–1004, 371bp) were cloned downstream of the T7 promoter in the pGEM-T easy vector (Promega). The sense and antisense RNA probes specific to the ChlI were prepared using DIG RNA Labeling Mix (Roche). Arrows indicate small RNAs of Y-sat. The 22-nt sequence complementarity between the ChlI and Y-sat is shown below each panel. A continuous 22-nt complementary sequence including G-U pairs is formed between the ChlI sense RNA and Y-sat sense RNA, but there are four mismatches in the region between the ChlI antisense RNA and Y-sat antisense RNA. (TIF) [file ppat.1002021.s002.tif]

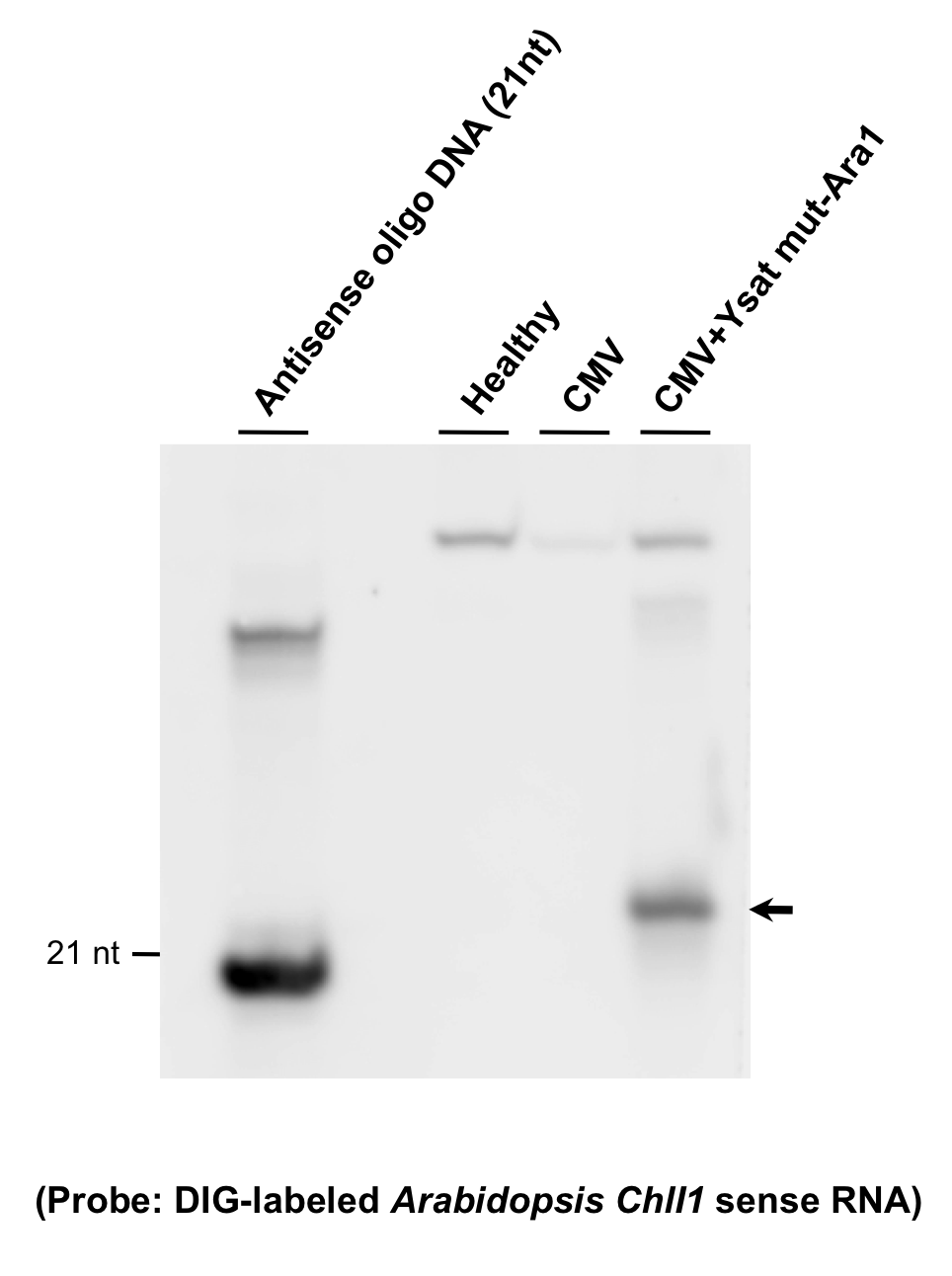

Supplement: Figure S3 — Northern blots of Y-sat mut-Ara1 small RNAs in Y-sat mut-Ara1-infected Arabidopsis. RNAs were prepared from Arabidopsis leaves infected with CMV or CMV+Y-sat mut-Ara1. For the RNA probe, the amplified ChlI1 fragments (750–1100, 351bp) were cloned downstream of the T7 promoter in the pGEM-T easy vector (Promega). The sense RNA probe specific to the Arabidopsis ChlI1 was prepared using DIG RNA Labeling Mix (Roche). Note that Arabidopsis ChlI1 sense RNA probe detected small RNAs from Y-sat mut-Ara1 (shown by an arrow) in the lane for Y-sat mut-Ara1-infected leaves. (TIF) [file ppat.1002021.s003.tif]

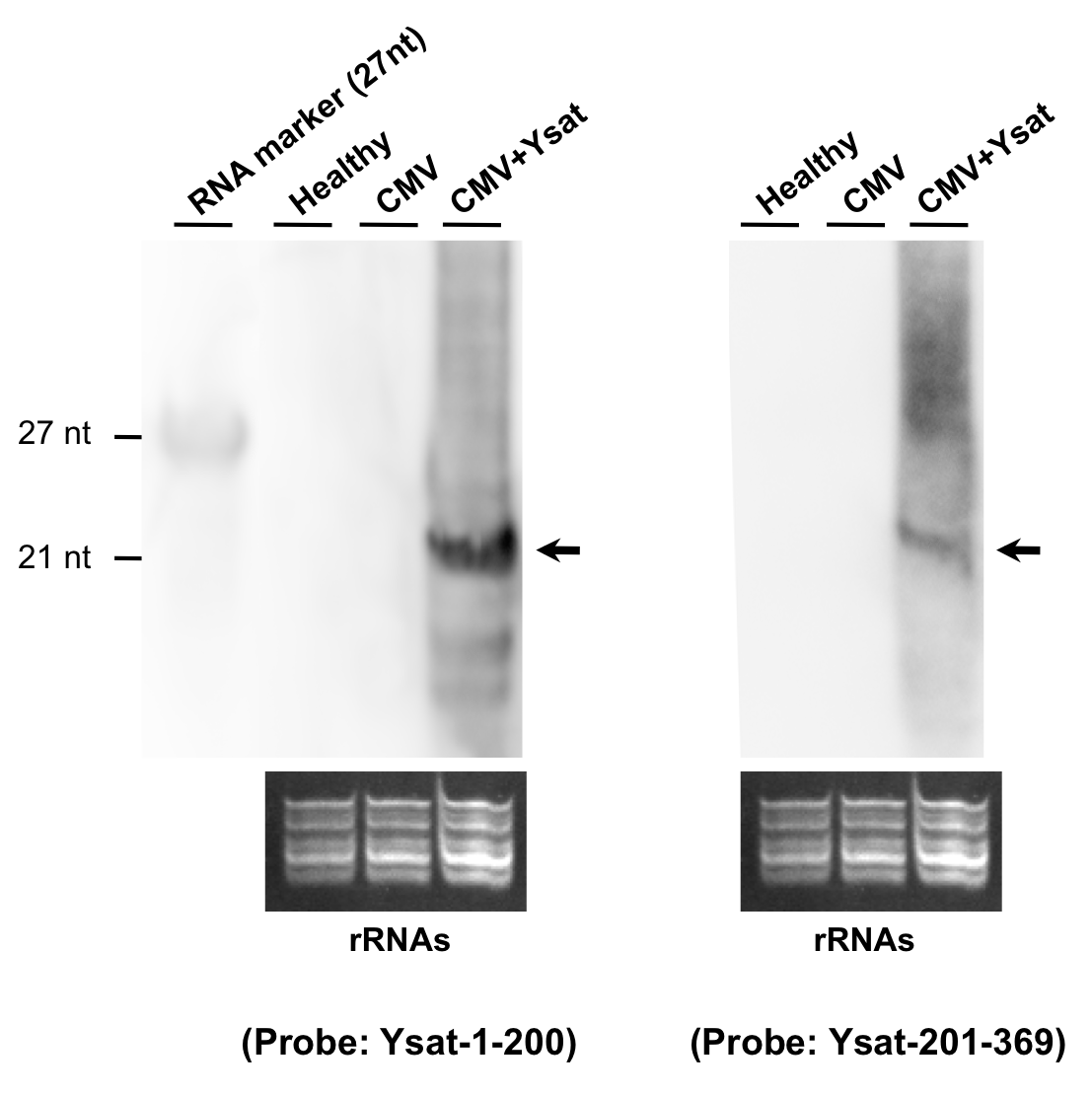

Supplement: Figure S4 — Confirmation of the relative abundance of Y-sat small RNAs by Northern blot hybridization. The small RNAs derived from the hot spots that were observed in the Y-sat small RNA profiles (Figure 5A) were detected and validated using DIG-labeled probes: Y-sat-1-200 and Y-sat-201-369. Y-sat-1-200 is complementary to the positions 1–200, and Y-sat-201-369 is complementary to the positions 201–369. The hybridization signals detected by Y-sat-1-200 (shown by an arrow in the left panel) were clearly stronger than those detected by Y-sat-201-369 (shown by an arrow in the right panel). These results support that the deep-sequencing approach reflects the hot spots identified for Y-sat small RNAs. (TIF) [file ppat.1002021.s004.tif]

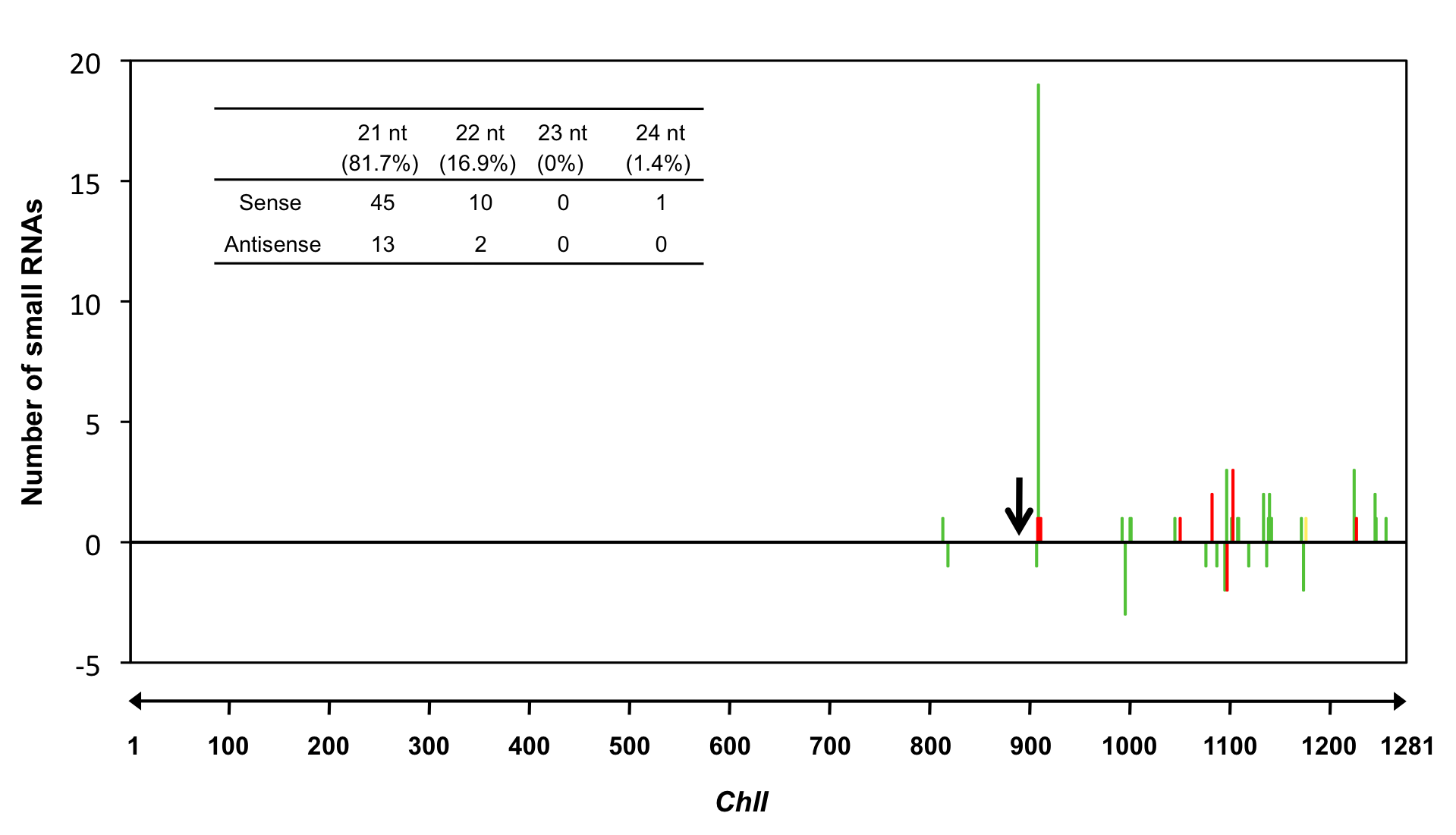

Supplement: Figure S5 — Deep-sequencing analysis of the ChlI small RNAs in N. benthamiana plants infected with CMV-Y and Y-sat. Location and frequency of the ChlI-derived small RNAs (21- to 24-nt) were mapped to the ChlI sequence in either sense (above the x-axis) or antisense (below the x-axis) orientation. Data from 21-, 22-, 24-nt small RNA are color-coded in green (21 nt), red (22 nt), and yellow (24 nt). Table in graph gives the number of small RNA reads and percentage of each size. Note that the small RNAs are mostly generated from the 3′ region downstream of the cleavage site indicated by an arrow. (TIF) [file ppat.1002021.s005.tif]
