# Supplementary material for: A Viral Satellite RNA Induces Yellow Symptoms on Tobacco by Targeting a Gene Involved in Chlorophyll Biosynthesis using the RNA Silencing Machinery
Source: PLoS Pathog. 2011 May 5;7(5):e1002021. doi: 10.1371/journal.ppat.1002021 (PMC3088725; doi:10.1371/journal.ppat.1002021)
Supplement: Table S1 — Genes downregulated in 16c:YsatIR 40% less than in 16c plants in microarray analysis. Among the 134 genes, 31 genes were chloroplast-related genes. (DOC) [file ppat.1002021.s006.doc]

Table S1. Genes downregulated in 16c:YsatIR 40% less than in 16c plants in microarray analysis. Among the 134 genes, 31 genes were chloroplast-related genes.

| No. | Ratio | GenBank ID | Unigene ID | Unigene Description |
| --- | --- | --- | --- | --- |
| 1 | 0.20 | EB451549 | Nta.1393 | Transcribed locus, moderately similar to NP_188245.1 water channel [Arabidopsis thaliana] |
| 2 | 0.22 | DW001361 | Nta.2585 | Transcribed locus, weakly similar to NP_566036.1 lipid transfer protein (LTP) family protein [Arabidopsis thaliana] |
| 3 | 0.25 | EB679796 | Nta.2591 | Transcribed locus, moderately similar to NP_197563.1 (GERMIN-LIKE PROTEIN 1); GER3 (GERMIN 3); oxalate oxidase [Arabidopsis thaliana] |
| 4 | 0.30 | DW001435 | Nta.202 | Transcribed locus, moderately similar to NP_194300.1 (BETA-HYDROXYLASE 1); beta-carotene hydroxylase [Arabidopsis thaliana] |
| 5 | 0.30 | CV018433 | Nta.9492 | Transcribed locus, moderately similar to NP_174286.1 (CHLOROPHYLL A/B BINDING PROTEIN 1); chlorophyll binding [Arabidopsis thaliana] |
| 6 | 0.33 | EB427492 | Nta.3273 | Transcribed locus, moderately similar to NP_849437.1 clathrin adaptor complexes medium subunit family protein [Arabidopsis thaliana] |
| 7 | 0.35 | DV162408 | Nta.5175 | Transcribed locus, strongly similar to NP_174286.1 CAB1 (CHLOROPHYLL A/B BINDING PROTEIN 1); chlorophyll binding [Arabidopsis thaliana] |
| 8 | 0.36 | DQ460158 | Nta.7488 | CDNA-AFLP fragment H-N_BC4M24-157 sequence |
| 9 | 0.40 | X52744 | Nta.7741 | Tabacco Cab40 mRNA for major chlorophyll a/b binding protein |
| 10 | 0.41 | Z14980 | Nta.6335 | N.tabacum Rca gene for ribulose bisphosphate carboxylase activase (pJQ4) |
| 11 | 0.42 | EB435365 | Nta.8552 | Transcribed locus, moderately similar to NP_199430.1 ornithine-oxo-acid transaminase [Arabidopsis thaliana] |
| 12 | 0.42 | DV159853 | Nta.6136 | Transcribed locus, moderately similar to NP_178969.1 AGT (ALANINE:GLYOXYLATE AMINOTRANSFERASE); alanine-glyoxylate transaminase/ serine-glyoxylate transaminase/ serine-pyruvate transaminase [Arabidopsis thaliana] |
| 13 | 0.43 | EB424655 | Nta.6354 | Chloroplast pigment-binding protein CP24 (Lhcb6) mRNA, complete cds; nuclear gene for chloroplast product |
| 14 | 0.43 | DV998840 | Nta.1361 | Transcribed locus, strongly similar to NP_200238.1 LHCB3 (LIGHT-HARVESTING CHLOROPHYLL B-BINDING PROTEIN 3); structural molecule [Arabidopsis thaliana] |
| 15 | 0.43 | Y12501 | Nta.4239 | Polyphenol oxidase |
| 16 | 0.43 | AF156369 | Nta.3950 | Clone PR27 alpha-2-HS-glycoprotein-like protein |
| 17 | 0.43 | U35111 | Nta.6161 | Rubisco activase precursor (Rca) |
| 18 | 0.44 | AM814121 | Nta.13595 | Transcribed locus, weakly similar to NP_194235.1 ACP4 (acyl carrier protein 4); acyl carrier [Arabidopsis thaliana] |
| 19 | 0.44 | EH613844 | Nta.2900 | Transcribed locus |
| 20 | 0.45 | CV016618 | Nta.9439 | Transcribed locus, strongly similar to NP_174286.1 CAB1 (CHLOROPHYLL A/B BINDING PROTEIN 1); chlorophyll binding [Arabidopsis thaliana] |
| 21 | 0.46 | EB681709 | Nta.2567 | Transcribed locus,moderately similar to NP_193016.1 PSAL (photosystem I subunit L) [Arabidopsis thaliana] |
| 22 | 0.46 | EB432397 | Nta.9782 | Transcribed locus,moderately similar to NP_850321.1 RCA (RUBISCO ACTIVASE); ADP binding / ATP binding / enzyme regulator/ ribulose-1,5-bisphosphate carboxylase/oxygenase activator [Arabidopsis thaliana] |
| 23 | 0.46 | CV016558 | Nta.9458 | Transcribed locus, moderately similar to NP_174286.1 (CHLOROPHYLL A/B BINDING PROTEIN 1); chlorophyll binding [Arabidopsis thaliana] |
| 24 | 0.47 | DW004730 | Nta.1472 | Transcribed locus, moderately similar to NP_188245.1 water channel [Arabidopsis thaliana] |
| 25 | 0.47 | DV999361 | Nta.1290 | Ribosomal protein L12 |
| 26 | 0.47 | EH616586 | Nta.8847 | Transcribed locus |
| 27 | 0.47 | AF014052 | Nta.7069 | Mg protoporphyrin IX chelatase (Chl H) |
| 28 | 0.48 | DV160428 | Nta.185 | CDNA-AFLP-fragment BSTT3-32-275, cultivar Bright Yellow 2, moderately similar to NP_173594.1 ATPDIL1-1 (PDI-LIKE 1-1); protein disulfide isomerase [Arabidopsis thaliana] |
| 29 | 0.48 | EB432718 | Nta.6294 | ATP synthase CF1 epsilon subunit |
| 30 | 0.48 | DV999800 | Nta.10595 | Transcribed locus, moderately similar to NP_850047.1 ribosomal protein L35 family protein [Arabidopsis thaliana] |
| 31 | 0.48 | U66266 | Nta.7727 | ORF |
| 32 | 0.49 | DV999224 | Nta.2554 | Transcribed locus, moderately similar to NP_172650.1 aminomethyltransferase, putative [Arabidopsis thaliana] |
| 33 | 0.50 | EB684021 | Nta.4664 | CDNA-AFLP-fragment BSTT41-1-280, cultivar Bright Yellow 2 |
| 34 | 0.50 | DV998999 | Nta.2663 | Transcribed locus, moderately similar to NP_201209.1 Photosystem I reaction center subunit (PsaN); calmodulin binding [Arabidopsis thaliana] |
| 35 | 0.50 | X62427 | Nta.7117 | N.tabacum mRNA (T23-2AF) for PSII 23-kDa polypeptide |
| 36 | 0.51 | EB680480 | Nta.6080 | Transcribed locus, strongly similar to NP_001078639.1 (GLUTAMINE SYNTHETASE 2) [Arabidopsis thaliana] |
| 37 | 0.51 | EB437069 | Nta.9965 | Transcribed locus, strongly similar to NP_174286.1 CAB1 (CHLOROPHYLL A/B BINDING PROTEIN 1); chlorophyll binding [Arabidopsis thaliana] |
| 38 | 0.51 | BP128401 | Nta.9382 | Transcribed locus |
| 39 | 0.52 | DW000008 | Nta.1474 | Transcribed locus, moderately similar to NP_192640.1APX4 (ASCORBATE PEROXIDASE 4); heme binding / peroxidase [Arabidopsis thaliana] |
| 40 | 0.52 | EB681488 | Nta.2976 | Photosystem I reaction center subunit X psaK mRNA, complete cds; nuclear gene for chloroplast product |
| 41 | 0.52 | EH617915 | Nta.9778 | Transcribed locus |
| 42 | 0.53 | EB680271 | Nta.6485 | Transcribed locus, strongly similar to NP_187204.1 RPT5A (REGULATORY PARTICLE TRIPLE-A ATPASE 5A); ATPase/ calmodulin binding [Arabidopsis thaliana] |
| 43 | 0.53 | AB074571 | Nta.6966 | POR2 mRNA for NADPH:protochlorophyllide oxidoreductase |
| 44 | 0.53 | EH618122 | Nta.8952 | Transcribed locus |
| 45 | 0.53 | EB424891 | Nta.10980 | Transcribed locus, moderately similar to NP_001078479.1 unknown protein [Arabidopsis thaliana] |
| 46 | 0.53 | DW003245 | Nta.11792 | NthisH3.1 mRNA for histone H3.1 |
| 47 | 0.53 | X55354 | Nta.7703 | N. tabacum mRNA for a photosystem II 23kDa polypeptide |
| 48 | 0.53 | DV160652 | Nta.10634 | Transcribed locus, weakly similar to XP_002266915.1 PREDICTED: hypothetical protein [Vitis vinifera] |
| 49 | 0.53 | CK720599 | Nta.5963 | Transcribed locus, moderately similar to NP_190910.1 PIP2A (PLASMA MEMBRANE INTRINSIC PROTEIN 2A); water channel [Arabidopsis thaliana] |
| 50 | 0.53 | EB683915 | Nta.10 | Transcribed locus, moderately similar to NP_201325.1 constituent of ribosome [Arabidopsis thaliana] |
| 51 | 0.54 | EB430863 | Nta.6658 | Transcribed locus, moderately similar to NP_194153.1 (VEIN PATTERNING) [Arabidopsis thaliana] |
| 52 | 0.54 | AF362948 | Nta.3804 | Endo-beta-1,4-glucanase precursor (Cel2) |
| 53 | 0.54 | EH623399 | Nta.8228 | Transcribed locus, moderately similar to NP_001065722.1 [Oryza sativa (japonica cultivar-group)] |
| 54 | 0.54 | BP129002 | Nta.3386 | Transcribed locus, moderately similar to NP_001067817.1 [Oryza sativa (japonica cultivar-group)] |
| 55 | 0.55 | AY554167 | Nta.2530 | Putative proline-rich protein |
| 56 | 0.55 | M14417 | Nta.7089 | Tobacco (N.tabacum) GapA mRNA encoding A-subunit of chloroplast glyceradehyde-3-phosphate dehydrogenase |
| 57 | 0.55 | EB451105 | Nta.10862 | Transcribed locus, moderately similar to NP_001077724.1 cytosolic factor (SEC14) / phosphoglyceride transfer protein [Arabidopsis thaliana] |
| 58 | 0.55 | EB432642 | Nta.8527 | Transcribed locus, weakly similar to NP_177492.1 MAP kinase [Arabidopsis thaliana] |
| 59 | 0.55 | AJ007789 | Nta.7270 | Geranylgeranyl reductase |
| 60 | 0.55 | EB451275 | Nta.5313 | Transcribed locus, moderately similar to NP_181013.1 (MULTIDRUG RESISTANCE-ASSOCIATED PROTEIN 2); ATPase, coupled to transmembrane movement of substances [Arabidopsis thaliana] |
| 61 | 0.56 | EB684135 | Nta.12740 | Transcribed locus, moderately similar to NP_001031102.2 SYP61 (SYNTAXIN OF PLANTS 61); SNAP receptor [Arabidopsis thaliana] |
| 62 | 0.56 | EB448302 | Nta.2581 | Transcribed locus, moderately similar to NP_564964.1 ATMINE1 (Arabidopsis homologue of bacterial MinE 1); protein binding [Arabidopsis thaliana] |
| 63 | 0.56 | DV160649 | Nta.3172 | Transcribed locus, moderately similar to NP_181933.1 COS1 (COI1 SUPPRESSOR1); 6,7-dimethyl-8-ribityllumazine synthase [Arabidopsis thaliana] |
| 64 | 0.56 | DV162447 | Nta.148 | Transcribed locus, moderately similar to NP_178585.1 LHCB2.1; chlorophyll binding [Arabidopsis thaliana] |
| 65 | 0.56 | EB431749 | Nta.12116 | Transcribed locus, strongly similar to NP_054505.1 ATP synthase CF1 epsilon subunit [Nicotiana tabacum] |
| 66 | 0.56 | BP533703 | Nta.11281 | Transcribed locus, weakly similar to NP_181329.2 unknown protein [Arabidopsis thaliana] |
| 67 | 0.56 | DV158750 | Nta.6858 | Transcribed locus, moderately similar to NP_195876.1 HTA12; DNA binding [Arabidopsis thaliana] |
| 68 | 0.56 | AJ719030 | Nta.9855 | CDNA-AFLP-fragment MBT14-320, cultivar Bright Yellow 2 |
| 69 | 0.56 | AJ717954 | Nta.4465 | CDNA-AFLP-fragment BSTT2-32-170, cultivar Bright Yellow 2 |
| 70 | 0.56 | EB424830 | Nta.226 | Transcribed locus, moderately similar to NP_566529.1 (FATTY ACID DESATURASE 5); oxidoreductase [Arabidopsis thaliana] |
| 71 | 0.56 | DV998829 | Nta.9417 | Transcribed locus, strongly similar to NP_191049.1 chlorophyll binding [Arabidopsis thaliana] |
| 72 | 0.56 | EB441390 | Nta.6607 | Transcribed locus, weakly similar to NP_188456.1 lipid transfer protein (LTP) family protein [Arabidopsis thaliana] |
| 73 | 0.56 | EB680782 | Nta.1316 | Transcribed locus, weakly similar to NP_566532.1 RanBP1 domain-containing protein [Arabidopsis thaliana] |
| 74 | 0.56 | EB680949 | Nta.2483 | Transcribed locus, weakly similar to NP_564046.1 unknown protein [Arabidopsis thaliana] |
| 75 | 0.56 | M60460 | Nta.3493 | Tobacco PR2 protein |
| 76 | 0.56 | EB431820 | Nta.9464 | Transcribed locus, strongly similar to NP_001078155.1 oxidase, peroxisomal, putative / glycolate oxidase, putative / short chain alpha-hydroxy acid oxidase, putative [Arabidopsis thaliana] |
| 77 | 0.56 | DV999315 | Nta.5383 | Transcribed locus, moderately similar to NP_175963.1 PSAG (PHOTOSYSTEM I SUBUNIT G) [Arabidopsis thaliana] |
| 78 | 0.57 | AF014053 | Nta.3589 | Mg protoporphyrin chelatase subunit (Chl I) |
| 79 | 0.57 | DV159953 | Nta.6507 | 1-deoxy-D-xylulose 5-phosphate synthase (dxs gene) |
| 80 | 0.57 | AY391749 | Nta.4114 | CMV 1a interacting protein 1 |
| 81 | 0.57 | DV162559 | Nta.6952 | Phosphomannomutase |
| 82 | 0.57 | X52743 | Nta.2705 | Tabacco Cab21 mRNA for major chlorophyll a/b binding protein |
| 83 | 0.57 | X80008 | Nta.4204 | N.tabacum mRNA for cytochrome b5 |
| 84 | 0.57 | EB680851 | Nta.6892 | Transcribed locus, weakly similar to NP_177177.2 RNA recognition motif (RRM)-containing protein [Arabidopsis thaliana] |
| 85 | 0.57 | EH615247 | Nta.9504 | Transcribed locus, strongly similar to NP_195876.1 HTA12; DNA binding [Arabidopsis thaliana] |
| 86 | 0.57 | AM819175 | Nta.14386 | Transcribed locus, moderately similar to NP_201299.2 ATATH13; transporter [Arabidopsis thaliana] |
| 87 | 0.57 | AY324804 | Nta.7435 | MRNA-binding protein precursor (csp41) mRNA, partial cds; nuclear gene for chloroplast product |
| 88 | 0.57 | X84225 | Nta.6986 | N.tabacum mRNA for precursor of photosystem II 22 kDa protein |
| 89 | 0.57 | DV159029 | Nta.1621 | Transcribed locus, weakly similar to NP_190719.1 unknown protein [Arabidopsis thaliana] |
| 90 | 0.58 | EH617111 | Nta.1813 | Transcribed locus, weakly similar to NP_189542.1 ion binding [Arabidopsis thaliana] |
| 91 | 0.58 | DW005082 | Nta.5795 | Transcribed locus, moderately similar to NP_563621.1 NC domain-containing protein-related [Arabidopsis thaliana] |
| 92 | 0.58 | DW003874 | Nta.11833 | Transcribed locus |
| 93 | 0.58 | EH614558 | Nta.1934 | Transcribed locus, weakly similar to NP_174202.1 constituent of ribosome [Arabidopsis thaliana] |
| 94 | 0.58 | EH624211 | Nta.9298 | Transcribed locus |
| 95 | 0.58 | DV999596 | Nta.6198 | Transcribed locus, moderately similar to NP_172405.1 CRB (CHLOROPLAST RNA BINDING); binding / catalytic/ coenzyme binding [Arabidopsis thaliana] |
| 96 | 0.58 | AJ719184 | Nta.5095 | CDNA-AFLP-fragment BSTT32-1-320, cultivar Bright Yellow 2 |
| 97 | 0.58 | AM833583 | Nta.14055 | Transcribed locus, moderately similar to NP_172009.1 galactosyltransferase family protein [Arabidopsis thaliana] |
| 98 | 0.58 | DV999353 | Nta.2953 | Ribosomal protein L27 |
| 99 | 0.58 | AM846775 | Nta.13757 | Transcribed locus |
| 100 | 0.58 | DV999634 | Nta.939 | Transcribed locus, moderately similar to NP_001078138.1 homogentisate solanesyltransferase [Arabidopsis thaliana] |
| 101 | 0.58 | EB682882 | Nta.12709 | Transcribed locus |
| 102 | 0.58 | AJ717873 | Nta.9864 | CDNA-AFLP-fragment BSTT12-4-420, cultivar Bright Yellow 2 |
| 103 | 0.58 | EB425604 | Nta.976 | Transcribed locus, weakly similar to NP_568650.1 protein [Arabidopsis thaliana] |
| 104 | 0.58 | EB425830 | Nta.11959 | Transcribed locus, weakly similar to NP_191002.1 AP3 (APETALA 3); DNA binding / transcription factor [Arabidopsis thaliana] |
| 105 | 0.58 | AF215852 | Nta.3724 | Hexose transporter (pGlcT) mRNA, partial cds; nuclear gene for chloroplast product |
| 106 | 0.58 | EB451056 | Nta.10635 | Transcribed locus, moderately similar to NP_564376.1 inositol-1(or 4)-monophosphatase [Arabidopsis thaliana] |
| 107 | 0.58 | EB442210 | Nta.9459 | Transcribed locus |
| 108 | 0.58 | EB427945 | Nta.12013 | Transcribed locus, weakly similar to NP_565348.1 lipid transfer protein (LTP) family protein [Arabidopsis thaliana] |
| 109 | 0.58 | EB438714 | Nta.12240 | Transcribed locus |
| 110 | 0.58 | EH617387 | Nta.8909 | Transcribed locus, weakly similar to NP_567196.1 auxin-responsive family protein [Arabidopsis thaliana] |
| 111 | 0.58 | EB449923 | Nta.4634 | Transcribed locus, weakly similar to NP_197744.1 AtCXE18 (Arabidopsis thaliana carboxyesterase 18); carboxylesterase [Arabidopsis thaliana] |
| 112 | 0.58 | DW003067 | Nta.11779 | Transcribed locus, moderately similar to NP_198391.1 SOS2 (SALT OVERLY SENSITIVE 2); kinase/ protein kinase [Arabidopsis thaliana] |
| 113 | 0.59 | EB683050 | Nta.12718 | Transcribed locus |
| 114 | 0.59 | EB679400 | Nta.2050 | Transcribed locus |
| 115 | 0.59 | AM844046 | Nta.5251 | Transcribed locus, moderately similar to NP_176908.2 exostosin family protein [Arabidopsis thaliana] |
| 116 | 0.59 | EH621433 | Nta.8629 | Transcribed locus |
| 117 | 0.59 | AM806128 | Nta.13817 | Transcribed locus |
| 118 | 0.59 | DW001775 | Nta.6611 | Transcribed locus, moderately similar to NP_172673.1 ELP (EXTENSIN-LIKE PROTEIN); lipid binding [Arabidopsis thaliana] |
| 119 | 0.59 | AJ719027 | Nta.3582 | CDNA-AFLP-fragment MBT14-150B, cultivar Bright Yellow 2 |
| 120 | 0.59 | EH664956 | Nta.7659 | NtEIG-E17 mRNA for glycine-rich protein |
| 121 | 0.59 | EB428295 | Nta.3359 | Transcribed locus, strongly similar to NP_195848.1 synbindin, putative [Arabidopsis thaliana] |
| 122 | 0.59 | AB112080 | Nta.7111 | 24K germin like protein |
| 123 | 0.59 | EH618639 | Nta.8985 | Transcribed locus |
| 124 | 0.59 | EH615352 | Nta.5392 | Transcribed locus, weakly similar to NP_565335.1 PSBX (photosystem II subunit X) [Arabidopsis thaliana] |
| 125 | 0.59 | DW002158 | Nta.11734 | Transcribed locus |
| 126 | 0.59 | BP129762 | Nta.3355 | Transcribed locus |
| 127 | 0.60 | DV999785 | Nta.11654 | Transcribed locus, strongly similar to NP_051055.1 photosystem II 44 kDa protein [Arabidopsis thaliana] |
| 128 | 0.60 | EB435916 | Nta.9779 | Transcribed locus, moderately similar to NP_193930.1 APR3 (APS REDUCTASE 3); adenylyl-sulfate reductase [Arabidopsis thaliana] |
| 129 | 0.60 | EB426408 | Nta.5340 | Transcribed locus, moderately similar to NP_568166.1 2-Cys Prx B (2-Cysteine peroxiredoxin B); antioxidant/ peroxiredoxin [Arabidopsis thaliana] |
| 130 | 0.60 | AM809066 | Nta.12950 | Transcribed locus |
| 131 | 0.60 | AM787148 | Nta.14305 | Transcribed locus |
| 132 | 0.60 | EB440419 | Nta.8591 | Transcribed locus, moderately similar to NP_566745.1ABC1 family protein [Arabidopsis thaliana] |
| 133 | 0.60 | AM824566 | Nta.13854 | Transcribed locus |
| 134 | 0.60 | Y18209 | Nta.3654 | Alpha-N-acetylglucosaminidase |
