# Supplementary material for: A Viral Satellite RNA Induces Yellow Symptoms on Tobacco by Targeting a Gene Involved in Chlorophyll Biosynthesis using the RNA Silencing Machinery
Source: PLoS Pathog. 2011 May 5;7(5):e1002021. doi: 10.1371/journal.ppat.1002021 (PMC3088725; doi:10.1371/journal.ppat.1002021)
Supplement: Text S1 — Supplementary materials and methods for the DNA microarray (Table S1) and two-dimensional electrophoresis experiments (Figure S1). (DOC) [file ppat.1002021.s007.doc]

**Text S1**

**Supplementary materials and methods**

DNA microarray experiments

We performed a microarray analysis using the oligonucleotide microarray (Filgen Array Nicotiana tabacum, Filgen), which contained 12832 tobacco probe sets. Fluorescent-dye-labeled antisense RNA was then synthesized by direct incorporation of Cy3-UTP or Cy5-UTP (GE Healthcare Bio-Science) using 20 μg of total RNA and an RNA Transcript SureLABEL Core Kit (TAKARA BIO INC.). The microarray was pre-hybridized for 1 h at 42 °C in a solution containing 5×SSC, 0.1% SDS, 400 mg/ml bovine serum albumin and 100 ng/μl salmon sperm DNA. The microarray was then washed three times in distilled water at room temperature for 2 min each time. Hybridization was performed for 16-20 h at 42 °C in a solution containing 5× SSC, 0.1% SDS, 10% formamide and heat-denatured, labeled cDNA. At room temperature, the microarray was then washed with 5× SSC and 0.05% SDS for 4 min, with 3× SSC and 0.05% SDS for 4 min, with 0.5× SSC for 1 min and with 2× phosphate-buffered saline (PBS) for 4 min. Fluorescence images of Cy3 and Cy5 dye channels were obtained using a GenePix 4000B scanner (Axon Instruments). For microarray data analysis, the program Array-Pro Analyzer Ver4.5 (Media Cybernetics) was used to determine the signal intensity of each spot. We normalized the biases in net intensity between the two fluorescent dye channels in a microarray by local regression (loess) normalization using the software. Analyzed data were selected using the MicroArray Data Analysis Tool (Filgen).

Two-dimensional electrophoresis experiments

Proteins were extracted from each equivalent leaf tissue (1 g) from 16C:YsatIR or 16c plants using the extraction buffer (50 mM Tris-HCl, 1 mM EDTA, 1 mM DTT, protease inhibitor cocktail, 4% PVPP). Purified proteins were first separated by isoelectric focusing electrophoresis using Immobiline dry strip pH3-10 (17-1234-01, Amersham Bioscience) and Ampholine pH3.5-10.0 for IEF (80-1125-87, Amersham bioscience). Then the proteins were further separated by SDS-PAGE (9–18% gradient gel) and stained with SYPRO Ruby (S21900, Invitrogen). We selected five spots that had markedly changed between 16c and 16c:YsatIR for LC-MSMS analysis. The analyzed proteins were identified by MASCOT search.
